# *GIPC2* is an endocrine-specific tumor suppressor gene for both sporadic and hereditary tumors of RET- and SDHB-, but not VHL-associated clusters of pheochromocytoma/paraganglioma

**DOI:** 10.1038/s41419-021-03731-7

**Published:** 2021-05-04

**Authors:** Yeqing Dong, Yongsheng Huang, Chengyan Fan, Liang Wang, Ran Zhang, Wenhua Li, Zhengguang Guo, Dong Wang, Zhi Zheng

**Affiliations:** 1 Institute of Basic Medical Sciences, Chinese Academy of Medical Sciences and School of Basic Medicine, Peking Union Medical College, Beijing, China; 2 Department of Urology, Peking Union Medical College Hospital, Peking Union Medical College and Chinese Academy of Medical Sciences, Beijing, China

**Keywords:** Cell-cycle proteins, Neuroendocrine cancer, Mechanisms of disease, Protein-protein interaction networks, Transcriptional regulatory elements

## Abstract

Pheochromocytoma/paraganglioma (PPGL) is an endocrine tumor of the chromaffin cells in the adrenal medulla or the paraganglia. Currently, about 70% of PPGLs can be explained by germline or somatic mutations in several broadly expressed susceptibility genes including *RET*, *VHL,* and *SDHB*, while for the remaining, mainly sporadic cases, the pathogenesis is still unclear. Even for known susceptible genes, how mutations in these mostly ubiquitous genes result in tissue-specific pathogenesis remains unanswered, and why *RET*-mutated tumors almost always occur in the adrenal while *SDHB*-mutated tumors mostly occur extra-adrenal remains a mystery. By analyzing 22 sporadic PPGLs using SNP 6.0 genotyping arrays combined with expression profiling of 4 normal and 4 tumor tissues, we identified *GIPC2*, a gene located at 1p31.1 with preferential expression in adrenal and inducible by adrenal glucocorticoid, as a novel putative tumor suppressor gene for PPGLs. Copy number deletion and *GIPC2* promoter hypermethylation but not *GIPC2* mutation, accompanied with reduced *GIPC2* expression, were observed in 39 of 55 PPGLs in our cohort. Examination of a published expression database consisting of 188 PPGLs found little *GIPC2* expression in Cluster 1A (SDHx-associated) and Cluster 2A (NF1/RET-associated) tumors, but less pronounced reduction of *GIPC2* expression in Cluster 1B (VHL-associated) and Cluster 2B/2C tumors. GIPC2 induced *p27*, suppressed MAPK/ERK and *HIF-1ɑ* pathways as well as cancer cell proliferation. Overexpressing GIPC2 in PC12 cells inhibited tumor growth in nude mice. We found GIPC2 interacted with the nucleoprotein NONO and both proteins regulated *p27* transcription through the same GGCC box on *p27* promoter. Significantly, low expression of both *GIPC2* and *p27* was associated with shorter disease-free survival time of PPGLs patients in the TCGA database. We found that PPGL-causing mutations in *RET* and in *SDHB* could lead to primary rat adrenal chromaffin cell proliferation, ERK activation, and *p27* downregulation, all requiring downregulating GIPC2. Notably, the RET-mutant effect required the presence of dexamethasone while the SDHB-mutant effect required its absence, providing a plausible explanation for the tumor location preference. In contrast, the PPGL-predisposing VHL mutations had no effect on proliferation and GIPC2 expression but caused p53 downregulation and reduced apoptosis in chromaffin cells compared with wild-type VHL. Thus, our study raises the importance of cortical hormone in PPGL development, and GIPC2 as a novel tumor suppressor provides a unified molecular mechanism for the tumorigenesis of both sporadic and hereditary tumors of Clusters 1A and 2A concerning SDHB and RET, but not tumors of Cluster 1B concerning VHL and other clusters.

## Introduction

Pheochromocytomas and paragangliomas (PPGLs) are catecholamine-secreting tumors that arise from chromaffin cells of the adrenal medulla and paraganglia, respectively. They can occur sporadically or as a part of different hereditary tumor syndromes, such as multiple endocrine neoplasia type 2 (MEN2) syndrome and Von Hippel-Lindau disease syndrome^1–3^. The germline and somatic mutations of about 20 genes can account for the pathogenesis of up to 70% of PPGLs^4–10^. The application of next-generation sequencing accelerated the discovery of driver susceptibility gene mutations. However, newly discovered mutations tend to occur in decreasing frequencies in PPGLs, suggesting that key driver mutations are close to being all found. Still, the remaining 30%, mostly sporadic cases do not harbor any known somatic mutations, indicating non-mutation-based mechanisms may be of importance for these cases.

Even for hereditary cases, the molecular mechanism of PPGL is far from clear. Few studies addressed how susceptibility gene mutations lead to PPGL pathogenesis in chromaffin cells. This is likely due to the lack of proper cellular models that can capture the phenotypes. For example, when PPGL-predisposing RET C634R mutation was introduced to PC12 rat pheochromocytoma cells, the cells underwent neuronal differentiation rather than proliferation^11^. On the other hand, for MEN 2A syndrome patients with the common RET C634R mutation, only about 50% develop pheochromocytoma^12^, suggesting the tumorigenesis may require additional genetic events^13^. Whole-genome scanning of PPGLs by array CGH (Comparative Genomic Hybridization) revealed that hereditary and sporadic PPGLs often harbor high-frequency deletions of 1p, 3pq, 11pq, 17p, 21q, especially 1p^14–17^. These suggest a new PPGLs-associated tumor suppressor gene may locate within 1p.

Previous studies of genome-wide transcription patterns classified PPGL into three major clusters: a pseudohypoxic/angiogenic cluster, including SDHx-related tumors (Cluster 1A) and VHL-related tumors (Cluster 1B), a kinase-signaling cluster, containing RET, NF1, and MAX-related tumors (Cluster 2A), and a Wnt-activated and Cortical Admixture cluster (Cluster 2B/2C))^17,18^. Interestingly, the majority of sporadic PPGLs classified into Cluster 2A^18^, suggesting shared common molecular pathways downstream with the RET/NF1 hereditary cases in this cluster.

The *p27* has been identified as a tumor suppressor gene relevant to PPGL. Both the *p27* knockout mice and the p27/p21 double knockout mice developed pheochromocytoma^19,20^. p27 mutation in rat results in MENX syndrome similar in phenotype and gene expression pattern to human pheochromocytoma^21,22^. Thus, p27/Rb signaling might be a diver pathway of PPGL. But how the known PPGL susceptibility genes regulate the p27/Rb signaling needs clarification.

In this study, using high-resolution microarrays and selecting for genes with preferential expression in adrenal, we identified a novel tumor suppressor gene *GIPC2*, that was inactivated in a majority of sporadic PPGL tumors due to copy number deletion and promoter hypermethylation. *GIPC2* is a gene located at 1p31.1, encoding a 315-amino-acid adaptor protein with a central PDZ domain for protein-protein interactions^23^. GIPC2 is primarily expressed in the adrenal, kidney, and colon and has been reported to be significantly downregulated in colon cancer, kidney cancer, and acute lymphocytic leukemia^23–25^. We present evidences that GIPC2 upregulates p27 and suppresses PPGL cell proliferation and tumor growth both in vitro and in vivo, and we propose a GIPC2-based mechanism through which sporadic and RET- and SDHB-related hereditary PPGLs develop.

## Results

We used SNP 6.0 arrays to analyze 22 sporadic PPGL tumors without common predisposing germline alterations and 14 matched blood samples, from a cohort of 55 PPGLs including 49 pheochromocytomas and 6 paraganglia (Supplementary File 1). In the analysis of copy number alterations, we noted significant copy number deletions on chromosomes 1p and 3q (Supplementary Fig. 1A), and identified 5507 genes with copy number deletion after narrowing down the minimal overlapping deletion intervals in 1p and in 3q. By analyzing 4 tumors and 4 normal adrenal medulla tissues on U133 plus 2.0 arrays, we identified 260 genes that were downregulated significantly in tumors. A total of 25 genes were found to have both copy number deletion and decreased expression (Supplementary Table 1). One of these genes, *GIPC2*, which is located at human chromosome 1p31.1^24^ was scored as preferentially expressed in human adrenal by the Tissue-Specific Genes Database (TISGED) analysis (http://bioinf.xmu.edu.cn:8080/databases/TiSGeD/index.html), and was selected for further study.

**Fig. 1:**
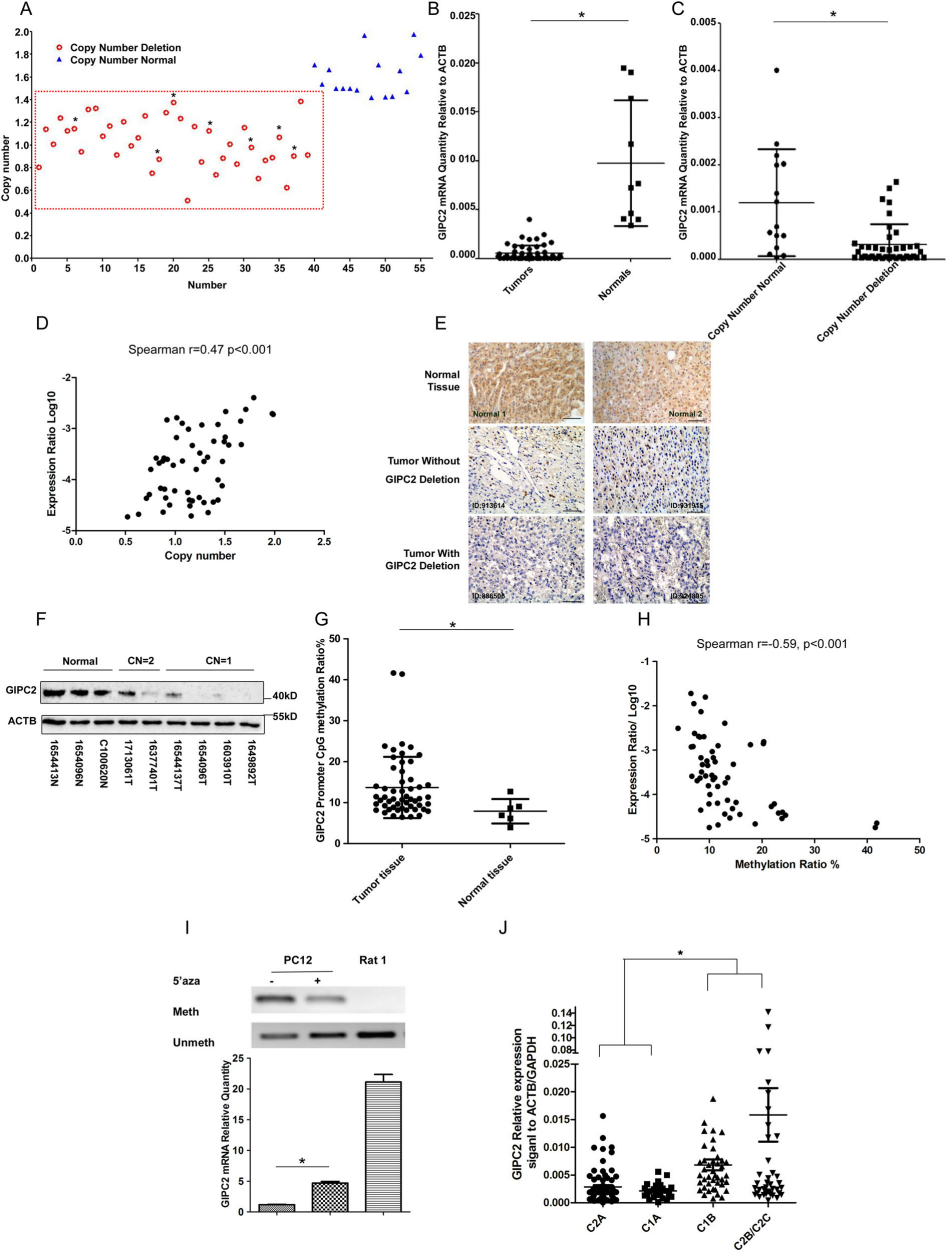
GIPC2 is a candidate tumor suppressor in sporadic PPGL. **A** The copy number variation of *GIPC2* in 55 PPGL tumors was verified by qPCR. The internal control gene was the human C2 gene. Copy number relative ratio <1.4 was determined as copy number deletion, while copy number relative ratio between 1.4 and 2.6 was considered as normal copy number. Tumor with germline or somatic RET mutation was indicated with an asterisk. **B** The mRNA level of *GIPC2* was measured in primary PPGL (*n* = 54) and normal medulla tissue (*n* = 10) by RT-PCR. **C** *GIPC2* mRNA levels were analyzed in copy number deletion tumors (*n* = 39) and normal copy number tumors (*n* = 15). **D** Correlation analysis between *GIPC2* copy number and its expression in PPGL samples (*n* = 54). **E** Immunohistochemistry of GIPC2 in the normal medulla and in PPGLs with or without *GIPC2* deletion. Scale bars represent 100 µm. Two representative samples were selected for each group. **F** Western blot of representative tumor samples using antibodies against GIPC2 and ACTB. **G** Methylation levels of *GIPC2* promoter were quantified by Sequenome EpiTYPER analysis in normal (*n* = 6) and PPGL samples (*n* = 53). **H** Correlation analysis between *GIPC2* methylation and its expression in PPGL samples (*n* = 59, including 6 normal samples). **I** Methylation-specific PCR assay for GIPC2 of DNA isolated from PC12 cells treated or untreated with 10 μM 5-AZA. PCR products labeled with “Meth” or “Unmeth” were generated by primers specific for methylated or unmethylated GIPC2. Relative gene expression of *GIPC2* was measured by qPCR, and rat adrenal medulla was examined as well for comparison. **J** *GIPC2* relative expression levels in PPGLs under different genetic subgroups. Data from E-MTAB-733 of the GEO database (http://www.ebi.ac.uk/arrayexpress/)^18^ on Affymetrix Human U133 plus 2.0 array, normalized by the geometric mean of *ACTB* and *GAPDH* expression levels.

We found 15 out of 22 samples had copy number deletion of *GIPC2* from SNP arrays data (an example in Supplementary Fig. 1B), and 39 tumors had copy number deletion from our cohort of 55 PPGL samples by qPCR, including all 7 RET-mutated PPGL (Fig. 1A and Supplementary File 1). Furthermore, *GIPC2* mRNA expression was significantly lower in PPGL tumors (*n* = 54, one sample showed mRNA degradation which was excluded) than normal adrenal medullas (*n* = 10) (Fig. 1B). The expression level of *GIPC2* decreased significantly in copy number deleted tumors compared with copy number normal tumors (Fig. 1C). There was a strong correlation between *GIPC2* mRNA expression and gene copy number (Fig. 1D). IHC staining of adrenal tissue sections demonstrated a moderate to high expression of GIPC2 in the nuclei and cytoplasm of normal medulla cells, low expression in tumors without *GIPC2* deletion, and no expression of GIPC2 in *GIPC2* deletion tumors (Fig. 1E). Western blot also confirmed the significantly reduced GIPC2 protein levels in tumor tissues (Fig. 1F).

We used Sanger sequencing to search for mutations in the six exons and exon/intron border regions of *GIPC2*. However, we found no germline or somatic mutations of GIPC2 in all 55 PPGL tumors and matched blood samples. Since aberrant promoter methylation was a well-recognized epigenetic mechanism involved in tumor suppressor gene silencing in cancers^25^, we determined the methylation levels of *GIPC2* promoter in PPGL samples by MALDI-TOF mass spectrometry (Sequenom EpiTYPER) and found significantly higher levels of methylation in the PPGL samples than normal tissues (Fig. 1G), as well as a negative correlation between *GIPC2* mRNA expression and methylation level (Fig. 1H). Methylation-specific PCR of 51 normal and PPGL samples confirmed that reduced *GIPC2* expression was associated with promoter hypermethylation (Supplementary Fig. 1C). In PC12 cells, a rat PPGL cell line, the expression of *GIPC2* increased 4.9-fold after treatment with DNA methyltransferase inhibitor 5-AZA (Fig. 1I). Similar results were observed in other cell lines (Supplementary Fig. 1D). An examination of a published gene expression database of 188 PPGL samples including 69 hereditary and 119 sporadic tumors (E-MTAB-733) (http://www.ebi.ac.uk/arrayexpress/)^18^ found that *GIPC2* expression was very low in the cases belonging to Clusters 1A and 2A, but relatively higher in the Cluster 1B and Cluster 2B/2C (Fig. 1J). This reduced expression appeared specific, as it was not observed for another GIPC family member nor for a neighboring gene on the chromosome (*PTGFR*, whose genome location adjoins *GIPC2*) (Supplementary Fig. 1E).

Taken together, we have found that a significant fraction of our sporadic PPGLs harbored GIPC2 genomic copy number loss and promoter hypermethylation, correlating with specifically reduced expression of GIPC2 protein.

### *GIPC2* suppresses cell proliferation in vitro and in vivo

**Fig. 2:**
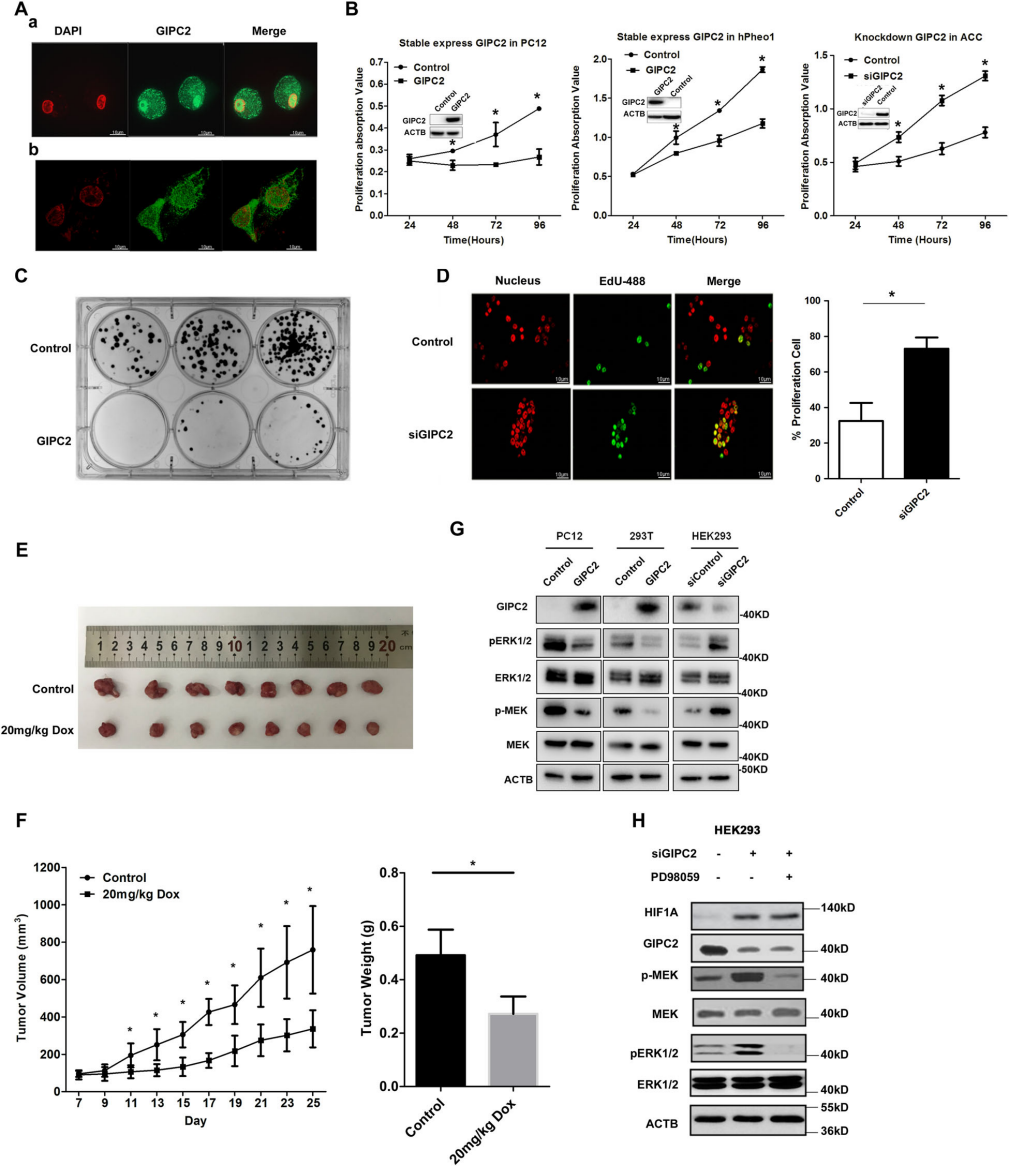
GIPC2 suppresses cell proliferation in vitro and in vivo. **A** Subcellular localization of GIPC2 protein in vivo. The distribution of endogenous GIPC2 was detected by immunofluorescent microscopy with anti-GIPC2 antibody in ACC cells (a) and hPheo1 cells (b). DAPI staining was included to visualize the cell nucleus. **B** PC12 cells and hPheo1 cells were infected with Tet-on lentiviruses systems carrying GIPC2 or vector, or ACC cells were transfected with the control or si-GIPC2. The growth curves of the cells in 96-well plates were measured with CCK-8 assay at an indicated hour. The efficiency of GIPC2 expression was verified by western blot (insets). **C** Stable cell lines of PC12-GIPC2 or control PC12 were maintained for 14 days in colony formation assay. **D** EdU imaging was performed and quantified in ACC cells which were transfected with the control or si-GIPC2. DAPI staining was included to visualize the cell nucleus. **E**, **F** PC12 cells infected with Tet-on lentiviruses carrying GIPC2 were inoculated into 4-week-old female BALB/c nude mice (3 × 10^6^ cells). When the subcutaneous tumors reached 80–100 mm^3^, mice were randomized into 2 groups and treated with saline as a control group or Doxycycline (20 mg/kg daily) to induce GIPC2 overexpression by intraperitoneal injection. The final tumor sizes were measured (**E**). The final tumor weights were determined and the tumor growth curve was plotted according to the tumor volume (**F**). Each bar represented the mean ± S.D. *n* = 8. **G** Protein lysates were prepared from PC12, 293T, and HEK293 cells with GIPC2 overexpression or knockdown and analyzed by western blot using antibodies against the indicated proteins. **H** Protein lysates were prepared from GIPC2-knockdown HEK293 cells with or without ERK inhibitor PD98059 treatment and analyzed by western blot using antibodies against the indicated proteins.

GIPC2 protein was normally expressed in the nucleus and the cytoplasm of cultured cells (Fig. 2A). The PDZ domain is important for the nuclear localization of GIPC2 and its cellular stability (Supplementary Fig. 2A–C).

To investigate whether *GIPC2* is a functional tumor suppressor gene, we performed cell proliferation assays, which showed that overexpression of GIPC2 significantly decreased proliferation in PC12 and hPheo1 cells (a cell line derived from a primary human pheochromocytoma^26^), while siGIPC2 significantly increased adrenal chromaffin cells (ACC) proliferation (Fig. 2B). Overexpression of GIPC2 in stably transfected cells inhibited the clone formation (Fig. 2C). The EdU staining assay indicated GIPC2 knockdown increased the cell proliferation in ACC cells (Fig. 2D). However, GIPC2 did not affect cell apoptosis (Supplementary Fig. 2D). Subsequently, a subcutaneous transplantation tumor model in nude BALB/c mice also confirmed the tumor suppression role of GIPC2 in tumor growth in vivo (Fig. 2E, F).

To explore the signaling mechanisms of GIPC2’s growth-inhibitory function, we investigated the MAPK/ERK, PI3K/AKT, and mTOR signaling pathways in GIPC2 overexpressing or knockdown cells. Significant downregulation of phospho-ERK1/2 and phospho-MEK was found in GIPC2 overexpressing cells while the upregulation of them was obtained in GIPC2-knockdown cells (Fig. 2G). However, the levels of phospho-AKT and phospho-mTOR exhibited no obvious changes (Supplementary Fig. 2E). HIF-1A was also upregulated by GIPC2 knockdown and this was independent of the MAPK/ERK pathway, as it was not blocked by MAPK/ERK inhibitor PD98059 (Fig. 2H). The above results indicate that GIPC2 suppresses cell proliferation, tumor growth, and inhibits MAPK/ERK and HIF pathways.

### GIPC2 upregulates *p27* transcription and arrests cell cycle

**Fig. 3:**
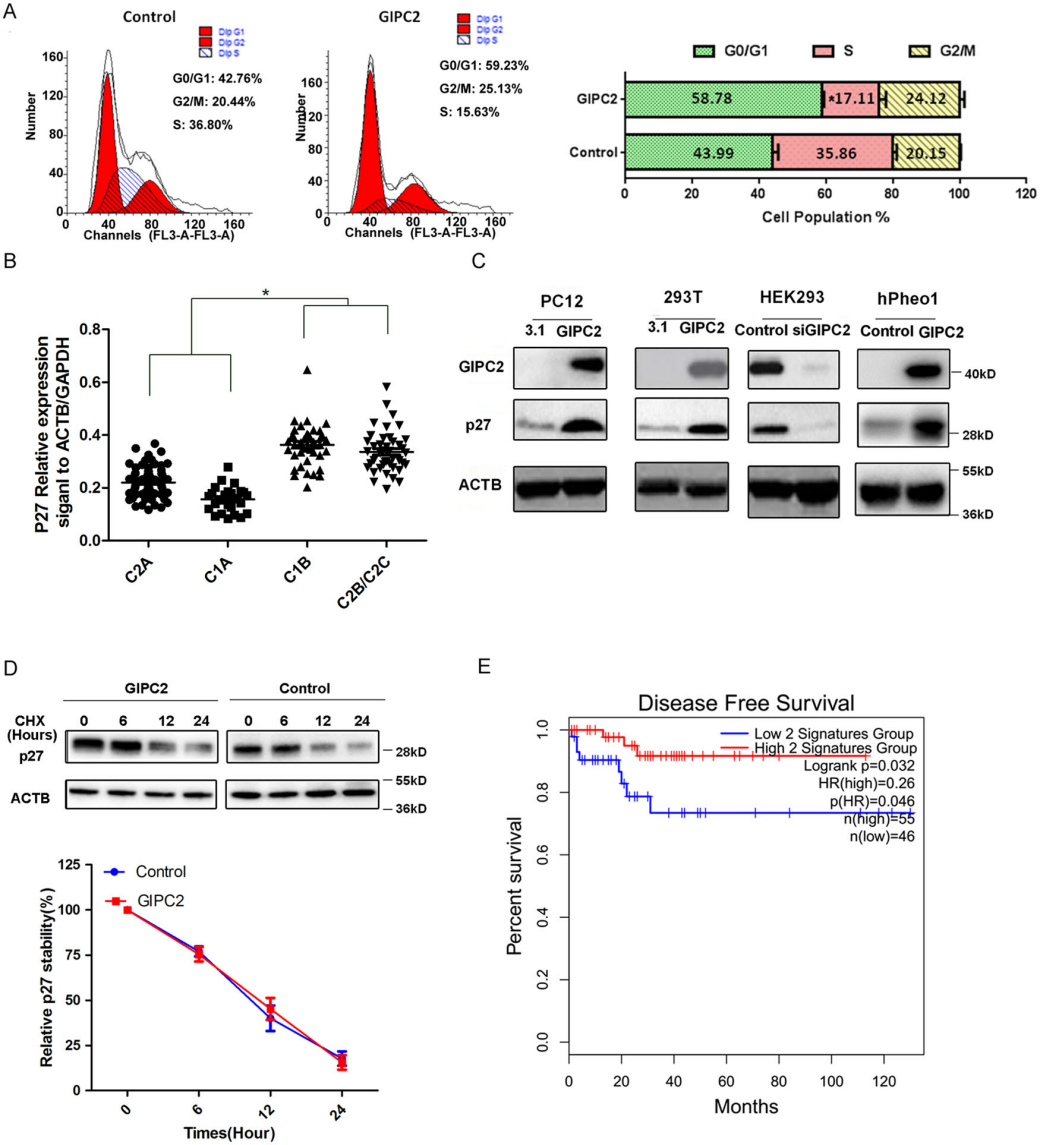
GIPC2 arrests cell cycle and upregulates the transcription of *p27*. **A** PC12 cells infected with Tet-on lentiviruses systems carrying GIPC2 was treated with Doxycycline for 72 h to induce GIPC2 overexpression prior to cell cycle analysis with ACCURI C6 flow cytometry using CFLOWPLUS and MODFIT software, respectively.　**B** The p27 relative expression signal in PPGL under different genetic subgroups. Data from GEO gene expression database (E-MTAB-733) (http://www.ebi.ac.uk/arrayexpress/)^18^.　**C** Total cellular proteins were prepared from PC12, 293T, HEK293, and hPheo1 cells with GIPC2 overexpression or knockdown, and p27 protein expression was analyzed by western blot.　**D** PC12 cells were treated with cycloheximide (CHX) in the presence or absence of overexpressed GIPC2. p27 protein levels were analyzed at 0, 6, 12, and 24 h time points after the addition of CHX by western blot and quantified. **E** Kaplan–Meier survival analysis of TCGA-PCPG data set for the disease-free survival time of the high 2 signatures (*GIPC2* and *p27*) group and the low 2 signatures group using the online tool GEPIA2 (gepia2.cancer-pku.cn/). The high 2 signatures group contains the samples with the top 30% of the two-gene (*GIPC2* and *p27)* signature expression values generated by GEPIA2 (*N* = 55), while the low 2 signatures group contains the samples with the bottom 25% of the two-gene signature expression values (*N* = 46). Disease-free survival time refers to the time until the occurrence of relapse, distant metastases, or positive regional lymph nodes.

To further characterize the effects of GIPC2, we performed flow cytometry analysis of cell cycle in PC12-Tet-GIPC2 stable cell lines, and found that overexpression of GIPC2 significantly blocked the transition from G0/G1 phase to S phase (Fig. 3A). Next, we examined the relationship between the expression of GIPC2 and the various cyclin-dependent kinase inhibitors using the data of 188 PPGLs gene expression database (E-MTAB-733). The expression pattern of p27 in the clusters was similar to that of GIPC2 in sporadic PPGL (Fig. 3B). Similar results were obtained in p18 (Supplementary Fig. 3A), but p16 and p21 did not show such a pattern (Supplementary Fig. 3B). Western blot confirmed that p27 was significantly increased after overexpression of *GIPC2* in PC12, 293T, and hPheo1 cells, while decreased when knocking down GIPC2 in HEK293 cells (Fig. 3C). It was reported that p27 protein level is primarily regulated post-translationally via proteasome-mediated degradation^27^. However, we found that GIPC2 did not affect the degradation of p27 (Fig. 3D). These results indicate that GIPC2 transcriptionally regulates *p27* to arrest the cell cycle.

### Clinicopathological relevance of GIPC2 and p27 in PPGL

To examine the clinical significance of the GIPC2-p27 axis in PPGL, we analyzed 182 PPGL cases from the TCGA-PCPG database, which did not distinguish sporadic or hereditary samples (portal.gdc.cancer.gov. Project ID:TCGA-PCPG). The mRNA expression of *GIPC2* was positively correlated with p27 (*r*(spearman) = 0.53) in this cohort (Supplementary Fig. 4A). Kaplan–Meier disease-free survival analysis found that the low expression of both *GIPC2* and *p27* had a lower disease-free survival time than the relatively high-expression group in PPGL patients (Fig. 3E), providing a clinicopathological relevance of *GIPC2*–*p27* axis in PPGL.

### GIPC2 physically interacts with NONO via PDZ domain

**Fig. 4:**
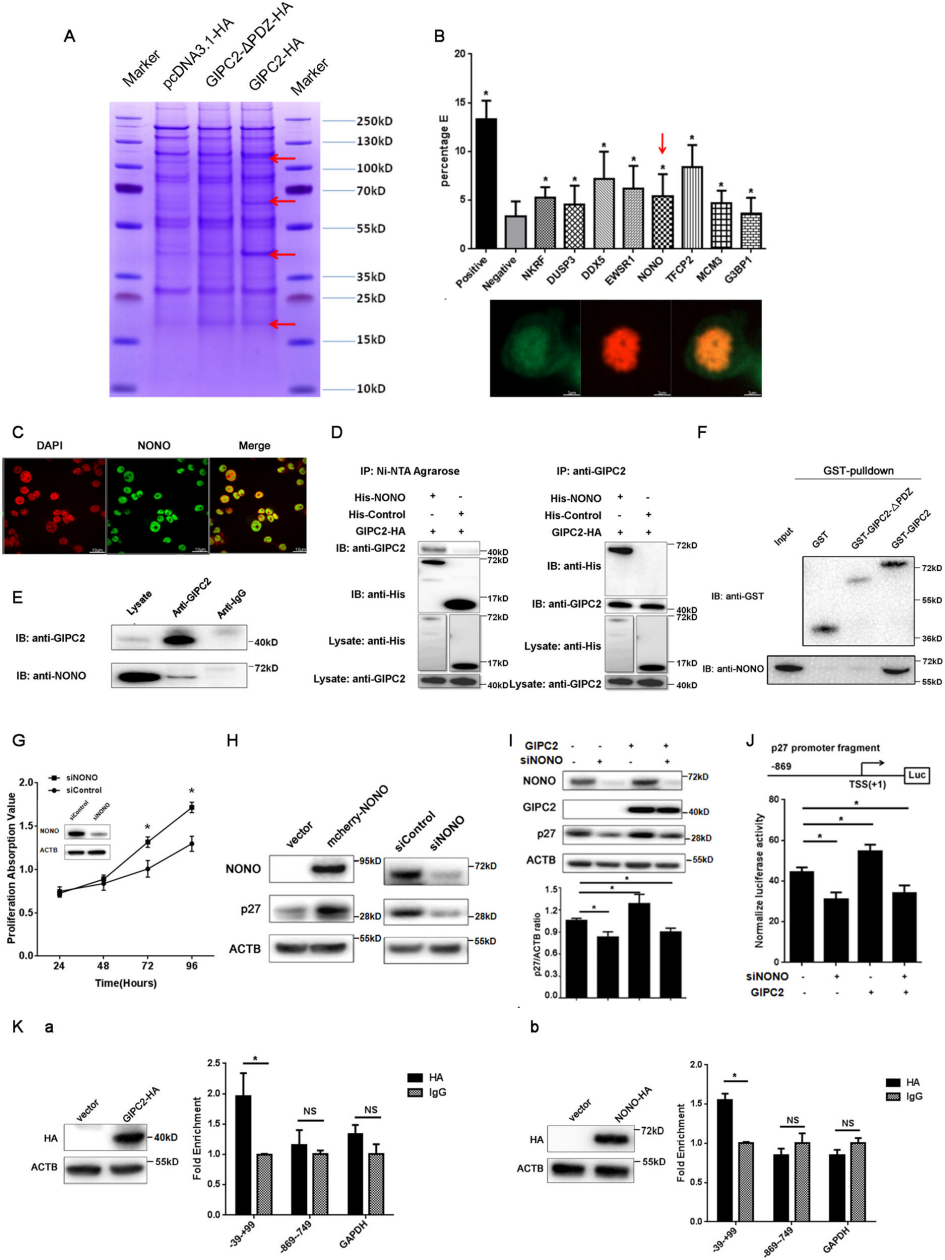
GIPC2 interacts with NONO and regulates the transcription of *p27*. **A** Immunoprecipitation-mass spectrometry analysis of GIPC2-associated proteins. Cellular extracts from 293T cells overexpressing GIPC2-HA, GIPC2-ΔPDZ-HA, or control 293T cells were immunoprecipitated with an anti-HA antibody. The eluates were resolved by SDS-PAGE and stained with coomassie brilliant blue. The four different protein bands (arrows) were retrieved and analyzed by mass spectrometry. **B** Interaction of GIPC2 with the candidate proteins. Top: Fluorescence resonance energy transfer (FRET) experiments were performed in PC12 cells co-transfected with GIPC2-AcGFP and mcherry-indicated proteins. Percentage E refers to the percentage of energy transfer efficiency. Bottom: Co-localization of GIPC2 and NONO protein in vitro. PC12 cells were co-transfected with GIPC2-AcGFP and mcherry-NONO and observed under a confocal microscope. Green represented GIPC2 and red represented NONO. **C** Subcellular localization of NONO protein in vivo. The distribution of endogenous NONO was detected by immunofluorescent microscopy with antibodies against NONO in PC12 cells. DAPI staining was included to visualize the cell nucleus. **D** Interaction of GIPC2 with NONO. Whole-cell lysates from 293T cells co-transfected with GIPC2-HA and His-NONO or control plasmids were prepared and immunoprecipitation was performed with Ni-NTA agarose and anti-GIPC2, respectively, followed by immunoblotting with antibodies against indicated proteins. (The WB diagrams of the “Lysate: anti-His” and “Lysate: anti-GIPC2” are identical in the left and right panel, as they are the same experiment of lysate, without IP, probed with the indicated antibody) **E** Whole-cell lysates from hPheo1 cells were immunoprecipitated with antibodies against GIPC2 or IgG followed by immunoblotting with the antibodies against the indicated proteins. **F** GST pull-down experiment. 293T lysates were incubated with bacterially expressed GST fused with either GIPC2 or GIPC2-ΔPDZ. Western blot analysis of the GST-fused proteins and interacting NONO protein were shown. **G** hPheo1 cells were transfected with the si-Control or si-NONO. The growth curves of the cells in 96-well plates were measured with CCK-8 assay. The efficiency of si-NONO knockdown in hPheo1 cells was verified by western blot. **H** Total cellular proteins were extracted from hPheo1 cells with NONO overexpression or knockdown and western blot was performed with indicated antibodies. **I** hPheo1 cells with stably transfected Tet-on lentiviruses systems carrying GIPC2 were transfected with si-NONO or si-Control for 24 h, together with or without tetracycline (3 μg/mL) for another 48 h. Western blot analysis was performed, to measure the relative level of p27 protein normalized by *ACTB*. **J** hPheo1 cells with stably transfected Tet-on lentiviruses systems carrying GIPC2 were transfected with si-NONO or si-Control, together with the indicated p27-luciferase reporter, and after 6 h treated with tetracycline (3 μg/mL) to induce GIPC2 overexpression. 48 h later, luciferase activity was measured. Relative luciferase activity was calculated as firefly luciferase activity divided by renilla luciferase activity and shown relative to the control (transfected with pGL-3-Basic vector). **K** Verification of the ChIP-qPCR results in hPheo1 cells. hPheo1 cells were transfected with GIPC2-HA (a) or NONO-HA (b). ChIP-qPCR analysis of the selected *p27* promoters was performed using antibodies against HA-tag or IgG. Results were represented as fold enrichment over control IgG with GAPDH as a negative control. The overexpression of GIPC2 or NONO was verified by western blot. Each point and bars of the pictures above represented the mean ± S.D. for triplicate experiments.

To further explore the molecular mechanism of tumor suppression by GIPC2, we used immunoprecipitation-mass spectrometry to identify GIPC2 interaction partners. The HA-tagged proteins were enriched by immunoprecipitation and coomassie bright blue staining were used to obtain 4 different bands (Fig. 4A). Mass spectrometry analysis identified 272 putative GIPC2 interacting proteins (Supplementary File 2). A series of nuclear-localized proteins were selected and verified for GIPC2 interaction using fluorescence resonance energy transfer (FRET) assay (Fig. 4B). Among them, NONO (p54nrb), a protein with known functions in regulating transcription^28,29^ was indeed localized in the nucleus of PC12 cells (Fig. 4C). The co-immunoprecipitation (Co-IP) experiment confirmed the interaction of NONO with exogenous or endogenous GIPC2 in vitro (Fig. 4D, E). To investigate the role of PDZ domain in this interaction, glutathione *S*-transferase (GST) pull-down experiments were performed with bacterially expressed GST fused to GIPC2 or a PDZ domain-deleted mutant. The results revealed that the PDZ domain of GIPC2 was necessary for its interaction with NONO (Fig. 4F). Altogether, these results establish an interaction between GIPC2 and NONO.

### GIPC2 regulates the transcription of *p27* through NONO

Based on the TCGA-PCPG database, the mRNA expression of *NONO* was positively correlated with p27 (*r*(spearman) = 0.79) and GIPC2 (*r*(spearman) = 0.55) (Supplementary Fig. 4A). Knockdown of NONO significantly accelerated cell proliferation in hPheo1 cells (Fig. 4G). Furthermore, overexpression of NONO increased the level of p27, whereas knocking down NONO decreased p27 (Fig. 4H). Thus, NONO can activate the expression of *p27* and inhibit cell proliferation, similar to GIPC2.

To evaluate whether NONO was required for the GIPC2 regulation of *p27*, hPheo1 cells with stable Tet-on GIPC2 (hPheo1-Tet-GIPC2) were transfected with siNONO or siControl, together with or without tetracycline (3 μg/mL) to induce GIPC2. Western blot analysis showed that GIPC2 was no longer able to promote p27 protein level when NONO was knocked down (Fig. 4I). Since GIPC2 had no DNA binding domain, it suggested that GIPC2 regulated the *p27* expression via NONO. To verify this, we cloned an 1198 bp fragment (−869/+328) from *p27* promoter. Luciferase reporter assay revealed that GIPC2 was able to activate the *p27* promoter activity, but not when NONO was knocked down (Fig. 4J). This suggests the critical role of NONO in GIPC2-regulated *p27* transcription.

In order to analyze the *p27* promoter binding sites for GIPC2/NONO, we established a series of luciferase reporter plasmids for truncated fragments of *p27* promoter from −2997, −869, −809, −482, −82, −34, +1, +37, +71, +90, +114, +179, to +328. Luciferase reporter assay revealed that GIPC2 could promote *p27* promoter activity by binding to the −34/+1 region of *p27* promoter (Supplementary Fig. 5A). Further, we used JASPAR network tool software (http://jaspar.genereg.net/) and PROMO network platform (http://alggen.lsi.upc.es/cgi-bin/promo_v3/promo/promoinit.cgi?dirDB=TF_8.3) to predict the DNA binding motif on the −34/+1 region, and built three mutation plasmids in the −34/+1 region of *p27* promoter (Supplementary Table 2). Results revealed that the GIPC2-responsive sites were both the AGGGG site and the GGCC box in *p27* promoter (Supplementary Fig. 5A). Similar analysis indicated that NONO was able to activate *p27* promoter activity, but only through GGCC motif (Supplementary Fig. 5B). Data from ChIP-qPCR experiments confirmed that both GIPC2 and NONO could bind to p27 promoter at the −39/+99 location (Fig. 4K). Collectively, these data support the notion that GIPC2 binds with NONO to regulate the transcription of *p27* through the GGCC box on *p27* promoter.

### The oncogenic effect of *RET* mutations on chromaffin cells requires the presence of glucocorticoid and is mediated by downregulating GIPC2

**Fig. 5:**
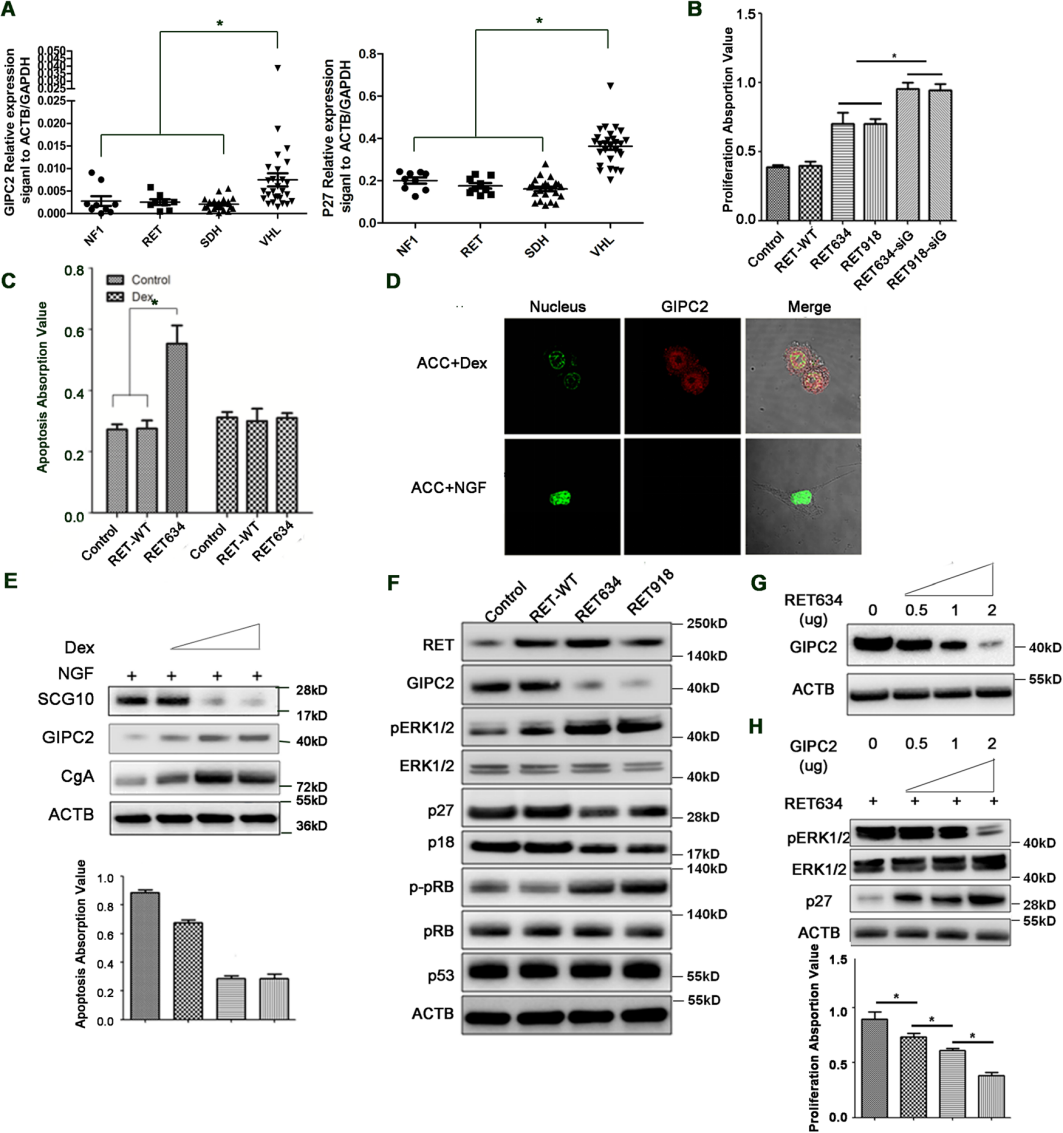
The oncogenic effect of *RET* mutations on chromaffin cells requires the presence of glucocorticoid and is mediated by downregulating GIPC2. **A** GIPC2 and p27 expressions were significantly reduced in hereditary *NF1-, RET-, SDH*-related tumors but not in hereditary *VHL*-related tumors in the data set of 188 PPGLs from the gene expression database (E-MTAB-733). **B** ACC cells in the presence of 10 μM Dexamethasone (Dex) in the media were transfected with RET634 (p.C634R) or RET918 (p.M918T) mutant plasmid or plasmid with additional GIPC2 knockdown. Cell proliferation was measured with CCK-8 assay at 48 h. **C** ACC cells were transfected with *RET* WT or RET634 mutant plasmid in the presence or absence of Dex. Apoptosis was assayed using Cell Death Detection ELISA Kit 48 h later. **D**, **E** ACC was treated with 100 ng/mL NGF or 10 μM Dex for 7 days. **D** Immunofluorescence of GIPC2 in ACC after 7 days. Green represents the nucleus, red represents GIPC2. **E** shows the protein changes and the apoptosis of ACC when treated with 100 ng/mL NGF in the absence or presence of 0.1, 1, and 10 μM Dex. **F** Western blot was performed in ACC cells in the presence of Dex with RET wild type or RET634/918 mutation overexpression, using antibodies against the indicated proteins. **G** GIPC2 protein levels with an increasing amount of RET634 mutant plasmid used during transfection of ACC cells in the presence of Dex were measured by western blot. **H** Western blot was performed to measure the protein levels of p27 and pErK1/2 under cotransfection of RET634 mutant and an increasing amount of GIPC2 plasmid in ACC cells in the presence of Dex. Proliferation was measured at 48 h.

While the preceding studies established the tumor-suppressing role of GIPC2 in sporadic PPGLs, we further explored whether GIPC2 had a role in *RET* mutation-related hereditary PPGL, since all 7 RET-mutated cases in our cohort had GIPC2 loss (Fig. 1A). In the data set of the 188 PPGLs mentioned earlier^18^, GIPC2 and p27 expression were significantly reduced in hereditary *RET*-related tumors as well (Fig. 5A). We first recapitulated PPGL using primary rat adrenal chromaffin cells (ACC). We demonstrated that in the presence of dexamethasone (Dex), an analog of the adrenal cortical glucocorticoid to which adrenal chromaffin cells are chronically exposed, ACC underwent proliferation instead of differentiation when transfected with either a MEN 2A-causing RET634 mutant or a MEN 2B-causing RET918 mutant (Fig. 5B), while no proliferation or differentiation was observed when ACC was transfected with wild type RET (Fig. 5B) or a FMTC-only *RET* mutant (RET768, data not shown), consistent with the in vivo MEN2 phenotype. A similar proliferative effect of RET mutant but not wild type can be observed in several cell lines in the presence of Dex (Supplementary Fig. 6A–C). In the absence of Dex, transfection of RET mutant but not wild type RET resulted in apoptosis in ACC (Fig. 5C), and in PC12 when the GIPC2 level was high (Supplementary Fig. 6D). The apoptosis required a specific PDZ domain of GIPC2 (Supplementary Fig. 6E). Treatment of ACC with nerve growth factor (NGF), which induced neuronal differentiation, significantly downregulated GIPC2 (Fig. 5D), while the addition of Dex maintained the chromaffin phenotype (Fig. 5D), induced the expression of GIPC2 and the endocrine cell marker CgA, but downregulated the neuronal marker SCG10 and the accompanying apoptosis (Fig. 5E). These suggest that GIPC2 plays specific roles only in the endocrine but not in neuronal lineage.

The proliferation accelerated in RET634/RET918-transfected cells when GIPC2 was knocked down (Fig. 5B), indicating opposing effects of GIPC2 and PPGL-causing RET mutations on proliferation. We found the level of GIPC2 and p27/p18 downregulated but phospho-ERK1/2 and phospho-RB significantly increased in RET634/RET918-transfected ACCs, while p53 level remained unchanged (Fig. 5F). The downregulation of GIPC2 by RET634 was dose-dependent (Fig. 5G). RET634’s effects on phospho-ERK and on p27 levels, and more importantly on the proliferation phenotype, appeared to be regulated via GIPC2, as these effects can be reversed dose-dependently by increasing GIPC2 (Fig. 5H). Together, these results suggest that the PPGL-causing *RET* mutation leads to chromaffin cell proliferation primarily via downregulating GIPC2.

### The oncogenic effect of *SDHB* mutation on chromaffin cells requires the absence of glucocorticoid and is mediated by downregulating *GIPC2*

**Fig. 6:**
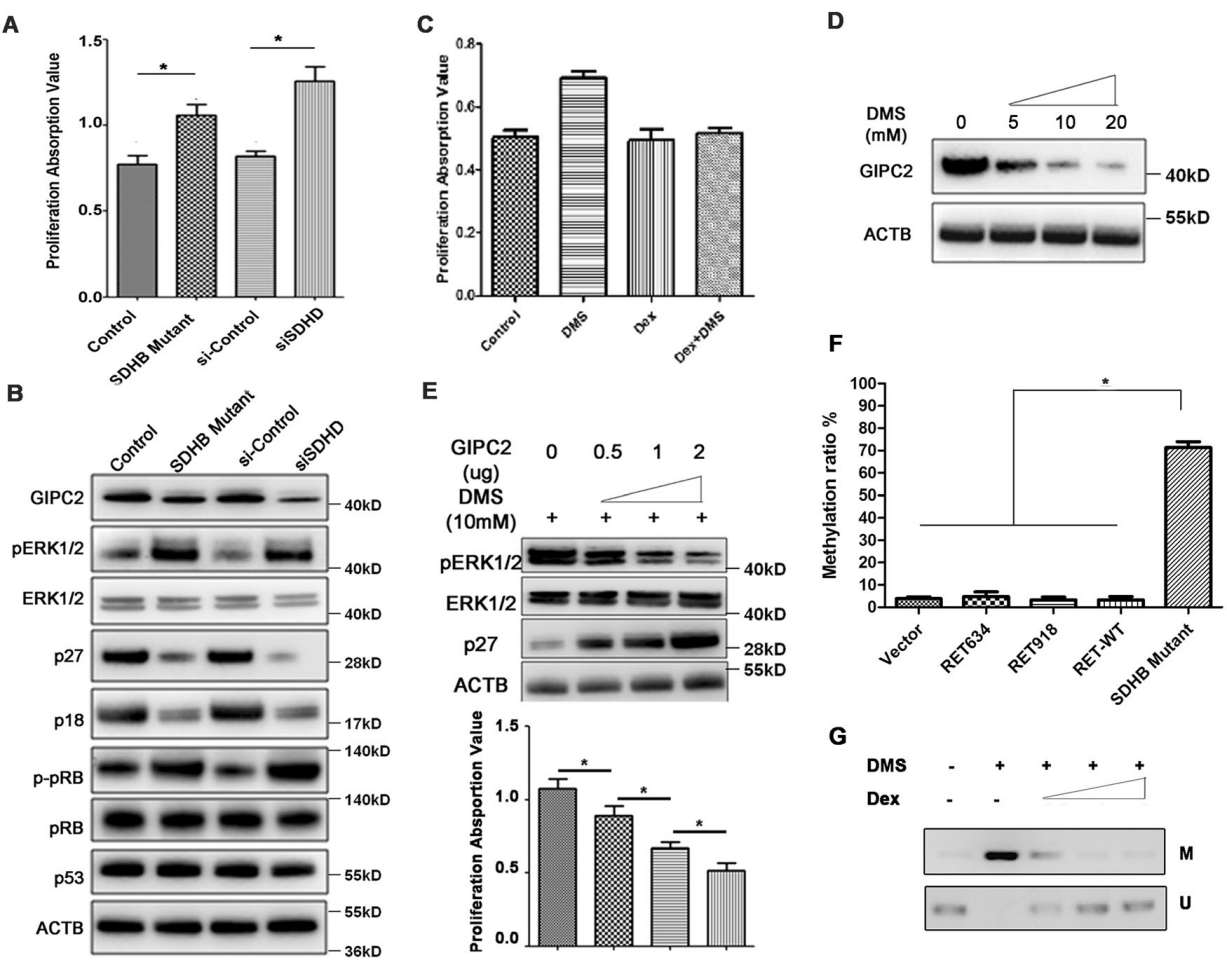
The oncogenic effect of *SDHB* mutation on chromaffin cells requires the absence of glucocorticoid and is mediated by downregulating GIPC2. **A** The proliferation of ACC cells overexpressing SDHB-mutant (p. R46Q) or si-SDHD^11^ was measured at 48 h. **B** Western blot was performed in ACC cells with SDHB-mutant overexpression or si-SDHD using antibodies against the indicated proteins. **C** ACC proliferation under 10 mM DMS and/or 10uM Dex treatment. **D** GIPC2 protein level with increasing DMS concentrations in ACC cells was measured by western blot. **E** Western blot was performed to measure the protein levels of p27 and pErK1/2 under 10 mM DMS stimulation and with an increasing amount of GIPC2 plasmid used during transfection of ACC cells. Proliferation was measured at 48 h. **F** GIPC2 methylation ratio as measured by EpiTyper assay in ACC transfected with RET and SDHB mutants. **G** Methylation-specific PCR assay was carried out to detect the methylation of GIPC2 promoter in ACC cells treated with 10 mM DMS and 0.1, 1, and 10 μM Dex.

We next asked whether *GIPC2* had a similar role in mediating the oncogenic effects of *SDHx* mutations in *SDHx*-related hereditary PPGL, since *SDHx*-related hereditary tumors also significantly downregulated GIPC2 and p27 (Fig. 5A). Mutations of the genes encoding succinate dehydrogenase subunits B (SDHB) and D (SDHD) are the most well-known causes of hereditary paraganglia^1,30^. The overexpression of a PPGL-causing *SDHB* mutation or the knockdown of wild type SDHD protein (both resulted in the increased intracellular concentration of oncometabolite succinate) led to ACC proliferation and corresponding changes of the downstream genes including *GIPC2*, *p27*, p18, phosphor-pRb, and phospho-*ERK*, similar to the *RET* mutant (Fig. 6A, B). As expected, direct treatment with a cell-permeable succinate analog dimethylsuccinate (DMS) also led to ACC proliferation; but contrary to the *RET* mutant case, the proliferating effect of DMS was abrogated in the presence of Dexamethasone (Fig. 6C). Without Dex, DMS behaved very similarly to the RET634 mutant in the presence of Dex in regulating downstream genes and proliferation phenotype (Figs. 5G, H and 6D, E). Thus, the oncogenic effect of PPGL-causing SDHB-mutation is also mediated by downregulating GIPC2.

Previous studies have reported that severe DNA hypermethylation occurred in *SDHx-*, particularly *SDHB*-related tumors^31^. We observed drastically increased *GIPC2* promoter methylation in chromaffin cells when transfected with *SDHB* mutant, but not *RET* mutants (Fig. 6F), suggesting different mechanisms of GIPC2 downregulation by *RET*- or *SDHB*-mutation. *GIPC2* promoter methylation can also be induced by DMS, but the methylation can be reversed by the presence of the increasing amount of Dex (Fig. 6G). In cell lines, the addition of DMS can also induce GIPC2 methylation as well as proliferation, while octyl-α-ketoglutarate, a membrane-permeating 2-KG analog, had opposite effects (Supplementary Fig. 7A, B).

### The PPGL-predisposing *VHL* mutations do not affect proliferation and *GIPC2* expression, but reduce chromaffin cell apoptosis via downregulating p53

**Fig. 7:**
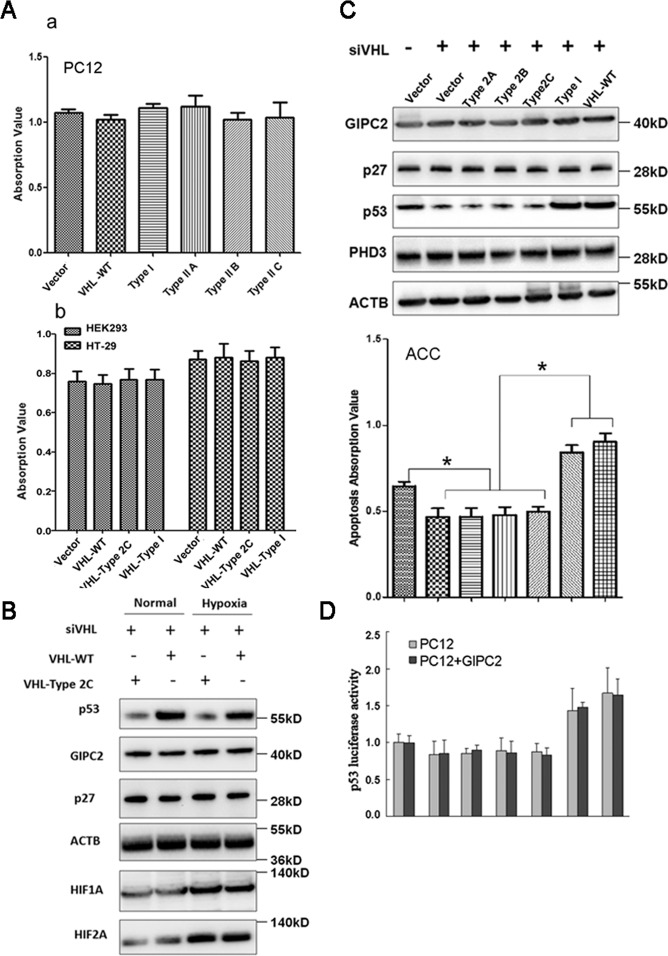
The PPGL-specific *VHL* mutation does not affect GIPC2 expression but reduces chromaffin cell apoptosis via downregulating p53. **A** Cell proliferation assay was performed in PC12 (a), HEK293, and HT-29 cells (b) with the overexpression of human VHL-type 2A (p.Y98H), Type 2B (p.W117R), Type 2C (p.L188V), and Type I (p.C162F) mutants and VHL-WT. **B** Under normal or hypoxia conditions, ACC was first transfected with a siRNA against rat VHL (siVHL)^11^ for 48 h, followed by transfection of VHL-WT or VHL-Type 2C mutant and was grown under low serum (1%) condition^11^. After 48 h western blot was performed using antibodies against the indicated proteins. **C** ACC was first transfected with si-VHL for 48 h, followed by transfection of VHL-WT or VHL mutants as indicated, and was grown under low serum (1%) condition. After 48 h Western blot was performed using antibodies against the indicated proteins (top panel). The cell apoptosis was determined using Cell Death Detection ELISA Kit (bottom panel). **D** PC12 cells and a PC12 line with stable overexpression of GIPC2 (PC12 + GIPC2) were treated as in **C**, together with a p53-luciferase reporter transfection. After 48 h, the cells were harvested and the luciferase activities were measured by the Dual-Luciferase Reporter Assay System (Promega, USA).

*GIPC2* and *p27* were not as significantly decreased in *VHL*-mutated tumors (Cluster 1B) as in Clusters A and 2A (Fig. 5A). The VHL syndrome mutations are classified into 2 types (VHL-type 1 and VHL-type 2). The VHL-type 1 mutation is not associated with PPGL and VHL-type 2C mutation is associated only with PPGL, while type 2A and 2B are associated with PPGL and other syndrome diseases^32^. Overexpression of VHL wild type and mutants did not induce proliferation (Fig. 7A). To reduce the effects of basal VHL activity, we first knocked down wild-type VHL in ACC before introducing various VHL constructs. In either normal or hypoxia conditions, the VHL 2C mutant relative to the wild type VHL did not affect the expression of GIPC2, p27, HIF-1A, and HIF-2A, but did reduce p53 (Fig. 7B). In fact, wild type and type I VHL mutant could stabilize p53 and induce apoptosis in ACC, but all type 2 VHL mutants failed to stabilize p53, and reduce the apoptosis of ACC (Fig. 7C). In addition, In PC12 cells, all type 2 mutants reduced the p53 activity compared with wild type and type 1 mutant, regardless of whether GIPC2 was overexpressed (Fig. 7D). The results indicate that p53 but not GIPC2 may be involved in the tumorigenesis of VHL-related PPGL.

## Discussion

We report the identification of a tumor suppressor gene, GIPC2 on chromosome 1p31.1, whose inactivation was associated with promoter hypermethylation and LOH in a majority of the sporadic PPGLs. We also provide evidence that the oncogenic effects on chromaffin cells by common RET- and SDH- mutations found in hereditary PPGL are mediated by downregulating GIPC2. This is the first high-frequency sporadic- and hereditary-PPGL tumor suppressor gene we know that does not involve mutation inactivation, and is therefore difficult to discover in previous next-generation sequencing attempts.

**Fig. 8:**
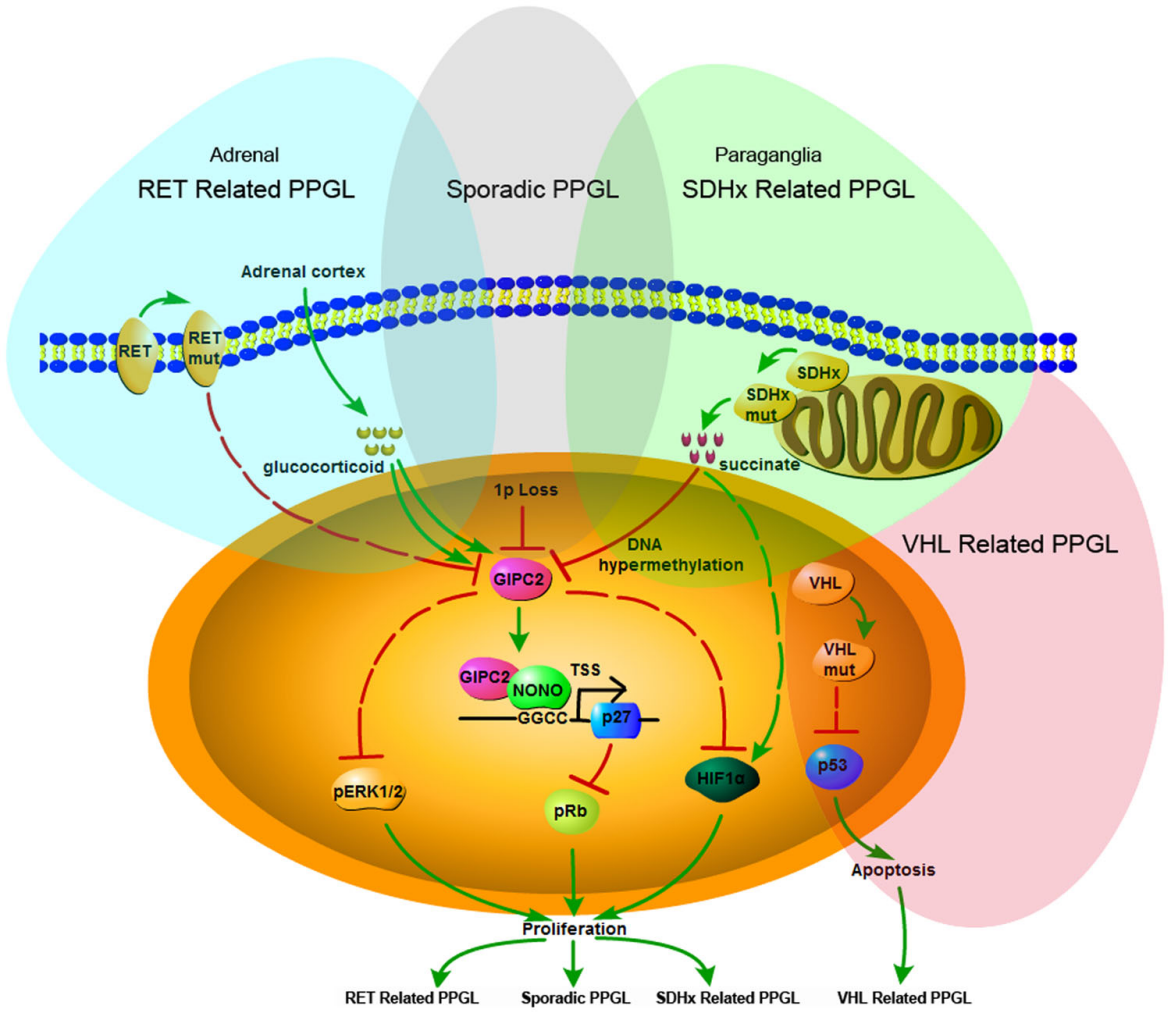
Proposed model of the molecular mechanism of RET-, SDHx-, and VHL-related hereditary as well as sporadic PPGL.

We propose a GIPC2-centered unified model for the development of both RET- and SDHB-associated sporadic and hereditary PPGLs (Fig. 8). Normally chromaffin cells in the adrenal and paraganglia are protected from PPGL by high expression of GIPC2. Germline mutations of RET and SDHB predispose the carrier to PPGL by downregulating GIPC2. Tumorigenesis begins when a sufficiently-low level of GIPC2 protein is reached, by either additional loss of GIPC2 by LOH of 1p including GIPC2 locus in predisposed cases, or 1p LOH and hypermethylation of GIPC2 locus in sporadic cases. Loss of GIPC2 resulted in p27 repression, activation of HIF-1alpha as well as the pERK pathways, proliferation of chromaffin cells, and possibly with the help of other 1p genes, oncogenic transformations leading to PPGL. We further identified the nucleoprotein NONO as a binding partner of GIPC2 directly regulating p27 transcription. A notable feature of GIPC2 is that it is an endocrine marker (Fig. 5D, E) in the sympathoadrenal lineage^33–35^ and therefore may confer the tissue specificity of the PPGL phenotype. Our model is only for Clusters 1A and 2A tumors and does not exclude the participation of other genes in the oncogenic transformation.

Our study also raises the importance of cortical hormones in PPGL development. Anatomically, the blood flow within the adrenal is directed centripetally from the cortex to the medulla^36^, and the close anatomical co-localization of the cortical and chromaffin cells^37^ may form the basis for a paracrine interaction. The addition of dexamethasone in chromaffin cell culture media kept the endocrine lineage and made it possible to recapitulate the PPGL phenotype (Fig. 5). It is well-known RET- and SDHx-related PPGLs occur almost exclusively in adrenal or extra-adrenal chromaffin cells, respectively^1–3^. We find glucocorticoid to be the environmental factor specifying the tumor location preference. Thus, RET-mutated chromaffin cells in the extra-adrenal (without glucocorticoid) undergo apoptosis and cannot form tumors, while in the adrenal they are protected by glucocorticoid from dying and proliferate (Fig. 5B, C). On the other hand, SDHB-mutated cells proliferate in extra-adrenal to form tumors by virtue of downregulating GIPC2 (Fig. 6), while in the adrenal the proliferation is countered by glucocorticoid-induced GIPC2 and tumor seldom forms (Fig. 5). Occasionally, SDHB-related tumors may arise in the adrenal, perhaps when GIPC2 induced by glucocorticoid fails to outweigh SDHx mutation-induced reduction.

Based on GIPC2’s role and expression level, the Cluster 1B (VHL-related) was distinct from Cluster 2A and 1A (RET- and SDHx-related respectively), a classification that was consistent with the cytogenetics observations of large numbers of PPGLs^16,17^, which showed the VHL-related tumors with chromosomal deletion patterns (mostly 3pq and 11p) that are distinct from those of RET- and SDHx-related tumors (mostly 1p and 3q). This classification is in contrast to molecular classifications based on expression profiling, which placed VHL-related tumors under “pseudohypoxia” cluster together with SDHx-related tumors^17,18^. Yet the evidence of a hypoxia mechanism of VHL-associated PPGL is sparse. It is well-known that type 2C VHL mutants, which predispose only to PPGL, are normal with respect to HIF regulation^38,39^, suggesting that a VHL target other than HIF is responsible for VHL-associated PPGL. VHL is known to associate with and stabilize p53^40^. We found the ability of a VHL mutation to predispose to PPGL is reflected by its ability to destabilize p53 and to reduce apoptosis (Fig. 7), suggesting that p53 may be the target mediating the effect of VHL. Given that expression profiling only establishes correlations between pathways and phenotypes, it is possible that the shared “pseudohypoxia” pathways with SDHx cluster may only reflect possible common functional phenotypes (e.g., oxygen sensing, or developmental stage) between the two groups of cells, and may have little to do with the driving mechanism of tumorigenesis.

## Materials and methods

### Patients and samples

Tumor samples were collected from 55 patients (49 pheochromocytoma and 6 paraganglia) at Peking Union Medical College Hospital, with the clinical information summarized in Supplementary File 1. Normal adrenal medulla tissues from nephrectomy patients were used as normal controls. Samples were collected with approval by the Institute ethics committee and informed consent from the patients.

### Microarray analysis

To detect copy number variation (CNV) in sporadic PPGL, Affymetrix genome-wide human SNP array 6.0 was used. The genomic DNA extraction, digestion, PCR, labeling, hybridization, and scanning were performed per the manufacturer’s instructions. Affymetrix HG-U133 plus 2.0 arrays were used to analyze mRNA expression. The total RNA extract, quality control, array hybridization, washing, and scanning were carried out as manufacturer’s instructions. Arrays were scanned and CEL files were imported to Partek Genomics Suite 6.0. The data were normalized using the Robust Multichip Averaging (RMA) algorithm and probe signal intensities were normalized by the MAS5 method.

### EpiTYPER methylation analysis

DNA methylation level was quantified with MassARRAY EpiTYPER assays (Sequenom, USA). Primers were designed using EpiDesigner (http://www.epidesigner.com). Bisulfite conversion of genomic DNA was performed using EZ DNA Methylation Kit. The average methylation ratios of the GIPC2 and control groups were calculated as the mean values of the CpG methylation rates and expressed as relative amount of methylation.

### Cell culture and reagents

PC12, HT-29, HEK293, and 293T cells were obtained from the National Infrastructure of Cell Line Resources, China. The hPheo1-Tet-GIPC2 or PC12-Tet-GIPC2 cells were stable cell lines custom made by Genecopoeia, China, and tetracycline or Doxycycline (3 μg/mL) was added to induce overexpression of GIPC2. Primary rat adrenal chromaffin cells were isolated from the adrenal glands according to the protocol of Domínguez et al.^41^, with the modification that only 5- to 7-days rats were used. All cells were cultured according to the guideline of American type culture collection (ATCC).

The primary antibodies against pERK1/2, ERK1/2, pMEK, MEK, p27, ACTB, p-pRB, p53, HA-Tag were purchased from Cell Signaling Technology (USA), antibodies against NONO, HIF1A, HIF2A, VHL were purchased from Abcam (UK), antibodies against p18, pRB were purchased from Santa Cruz (USA), GIPC2, and RET antibodies were purchased from LSBio (USA) and Abbiotec (USA) respectively. GIPC2 siRNA were purchased from Dharmacon. SDHD and VHL siRNAs^11^ were synthesized by Invitrogen. For the RNAi experiment, siTran1.0 Reagent (Origene, USA) was used according to the manufacturer’s instructions.

### Tumor formation in nude mice

For the xenograft model, 4-week-old female BALB/c nude mice were injected subcutaneously with 3 × 10^6^ PC12-Tet-GIPC2 cells. When the volume of tumors reached 80–100 mm^3^, mice were randomized into 2 groups (*n* = 8 per group). Group 1 was treated with saline as control, and group 2 was treated with Doxycycline (20 mg/kg) to induce GIPC2 expression by intraperitoneal injection every day. The tumor length (L) and width (W) were measured every 2 days using calipers and then the tumor volume was calculated (tumor volume =0.52 × length × width^2^). Once the diameter of the tumor exceeded 15 mm, the mice were euthanized. All animal experiments were approved by the animal ethics committee of the Chinese Academy of Medical Sciences.

### Immunoprecipitation-mass spectrometry

HEK 293T cells were lysed and centrifuged. The supernatants were incubated with primary antibody at 4 °C overnight and then captured with protein A+G beads (Beyotime, China) or Ni-NTA beads (GE, USA). The proteins labeled with HA were enriched by immunoprecipitation, and the reaction products were subjected to SDS-PAGE electrophoresis and coomassie blue staining. Different bands in the experimental groups were excised, and in-gel tryptic digested. The digested peptide was analyzed as previously described^42^ by LC–MS/MS on a Triple TOF 5600 mass spectrometer (AB Sciex, Framingham, MA, USA). MS/MS data were searched against the SwissProt human database (20227 entries) using Mascot (version 2.4.01; Matrix Science, London, UK), and processed using Scaffold software (version 4.0.7; Proteome Software Inc., Portland, OR, USA).

### Fluorescence resonance energy transfer

Cells were plated into 8-well culture dishes coated with poly-l-lysine and co-transfected with pairs of expression constructs. After transfection for 48 h, fluorescence signals were collected. All images were acquired using UltraVIEW VoX- 3D Live Cell Imaging System and analyzed using velocity 6.1 software.

### GST pull-down assay

The constructed plasmids were transformed into BL21 competent cells and a large amount of GST, GIPC2-GST, and GIPC2-△PDZ-GST protein was induced by adding IPTG at room temperature overnight. Then, the proteins were collected after ultrasonic crushing, and mixed with GST-beads (GE, USA), and incubated at 4 °C overnight. The mixture was centrifuged, washed, added 293T lysates, and washed again. After discarding the washes, an equal volume of 2× SDS gel-loading buffer was added to the beads. The mixture was boiled for 5 min in a water bath and the supernatants were collected for SDS-PAGE and western blot analysis.

### Dual-luciferase reporter assay

The wild type, truncated, and mutant *p27* promotor were amplified from genomic DNA template using corresponding PCR primers shown in Supplementary Table 3, and cloned into pGL3-basic vector (Promega, USA). After transfection of Reporter and pRL-TK (Renilla) (Promega, USA) or target plasmids for 6 h, the hPheo1-Tet-GIPC2 cells were treated with tetracycline (3 μg/mL) for another 42 h. The cells were then harvested and the luciferase activities measured by the Dual-Luciferase Reporter Assay System (Promega, USA).

### Chromatin immunoprecipitation (ChIP)

ChIP experiments were performed using Simple ChIP Kit (Cell Signaling Technology, USA). Anti-HA antibody was used to precipitate the DNA–protein complex. Normal IgG provided in Simple ChIP Kit was used as the negative control. Purified DNA obtained from the precipitate was used as the template and PCR was conducted using a pair of primers (Supplementary Table 3), which were designed based on the different NONO-binding sites of the *p27* promoter.

### Statistical analysis

The data are presented as the mean ± SD of at least three independent experiments. Data were analyzed using the one-way ANOVA method or Student’s *t*-test, *P < 0.05 was considered statistically significant. GraphPad Prism version 6.00 was used for statistic analysis.

## Supplementary information

Supplementary Figures

Supplementary Tables

Supple. File 1. Clinical information of the PPGL samples

Supple. File 2. Protein identification results of the IP-MS experiment

## Data Availability

The authors declare that all relevant data of this study are available from the corresponding author on reasonable request.
