# Supplementary material for: GIPC2 is an endocrine-specific tumor suppressor gene for both sporadic and hereditary tumors of RET- and SDHB-, but not VHL-associated clusters of pheochromocytoma/paraganglioma
Source: Cell Death Dis. 2021 May 4;12(5):444. doi: 10.1038/s41419-021-03731-7 (PMC8096975; doi:10.1038/s41419-021-03731-7)
Supplement: Supplementary file 1 — Supplementary Figures [file 41419_2021_3731_MOESM1_ESM.docx]

**Supplementary Figures**

**
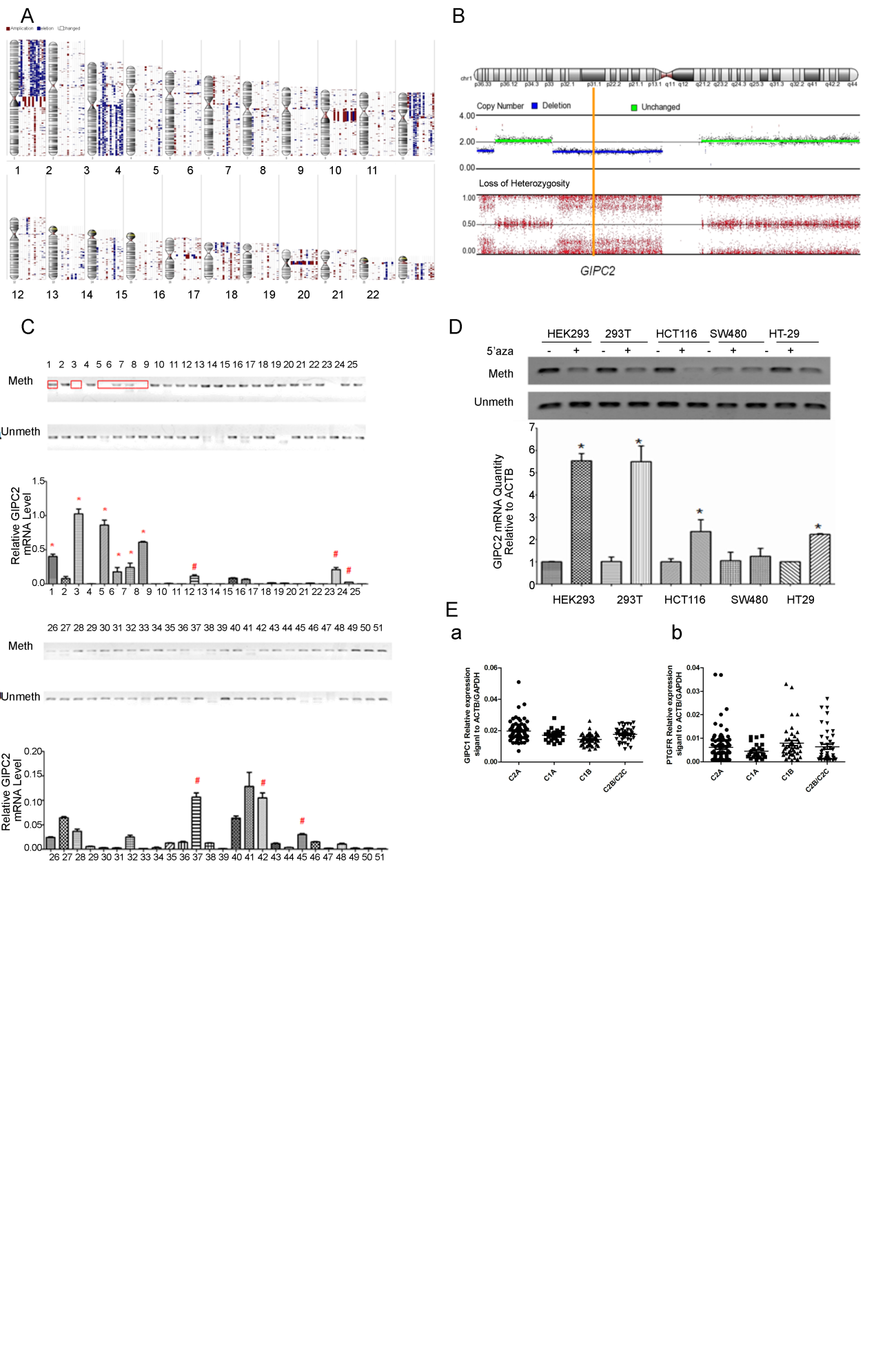
**

**Supplementary Fig.1 GIPC2 is a novel tumor suppressor in sporadic PPGL.**

**A** Copy number variation of 14 PPGLs using SNP 6.0 array and Partek6.0 software. Red showed copy number amplification and blue showed copy number deletion. **B** Sample 1684676 as an example showing copy number deletion and LOH around the GIPC2 locus. **C** Methylation levels of GIPC2 promoter as assayed by methylation-specific PCR, and the corresponding GIPC2 expression levels as assayed by qPCR, were shown for normal and PPGL samples with or without GIPC2 deletion. PCR products labeled with ‘Meth’ or ‘Unmeth’ were generated by primers specific for methylated or unmethylated DNA. Each *bar* represented the mean ± S.D. for triplicate experiments. * indicated normal samples, # indicated PPGL samples without GIPC2 deletion and others were GIPC2 deletion PPGL. **D** Methylation-specific PCR assay on DNAs isolated from 5-AZA treated or untreated cell lines as indicated. Corresponding mRNA levels by qPCR were shown below. **E (a)** *GIPC1* and (**b)** *PTGFR* relative expression signal in PPGL under different genetic subgroups. The data were from E-MTAB-733(http://www.ebi.ac.uk/arrayexpress/) on Affymetrix Human U133 plus 2.0 array, normalized by geometric mean of ACTB and GAPDH.


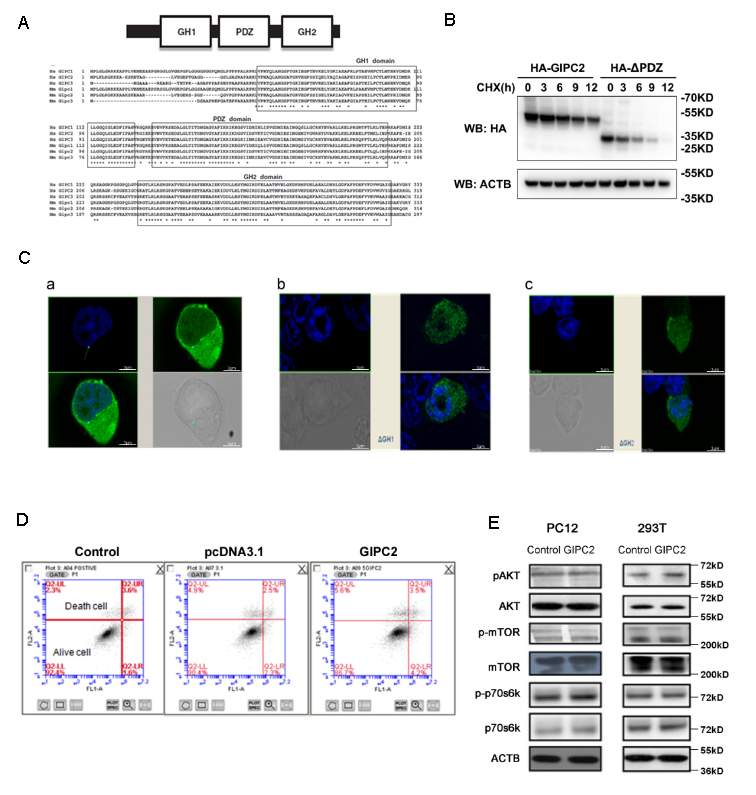


**Supplementary Fig.2 PDZ domain is important for stability and cellular localization of GIPC2 protein.**

**A** Domain architecture of GIPC family proteins consisting of a GH1 domain, a PDZ domain and a GH2 domain. **B** GIPC2 and GIPC2-∆PDZ were transfected into HEK293T cells, and after 12 hours cells were treated with 100ug/ml cycloheximide (CHX). Samples were harvested at 0, 3, 6, 8, and 12 h time points after the addition of CHX. Protein lysates were analyzed by western blot using antibodies against the indicated proteins. **C** GIPC2-∆PDZ(a), GIPC2-∆GH1(b) and GIPC2-∆GH2(c) location in PC12 cells. GIPC2 variants were transfected into PC12 cells and detected by immunofluorescence with HA antibody. Blue represented DAPI and green represented GIPC2 variants. **D** PC12 cells transfected with GIPC2 or control were harvested for apoptosis analysis on a ACCURI C6 flow cytometry through PI/AnnexinV kit . **E** Protein lysates were prepared from PC12 and 293T cells which were transfected with GIPC2 or control and analyzed by western blot using antibodies against the indicated proteins.


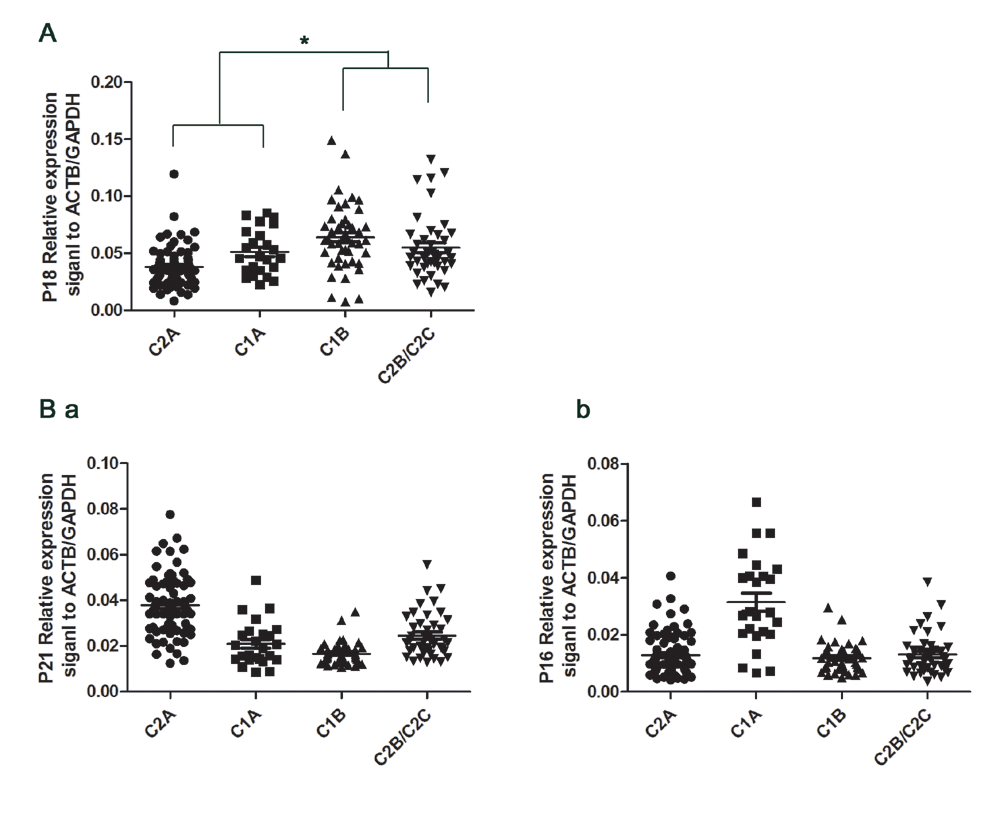


**Supplementary Fig.3 The *p18, p16* and *p21* relative expression in PPGL.**

**A** The *p18* relative expression signal in PPGL under different genetic subgroups. **B** The *p16* (a) and *p21* (b) relative expression signal in PPGL under different genetic subgroups. All the data were derived from GEO database (E-MTAB-733).

**
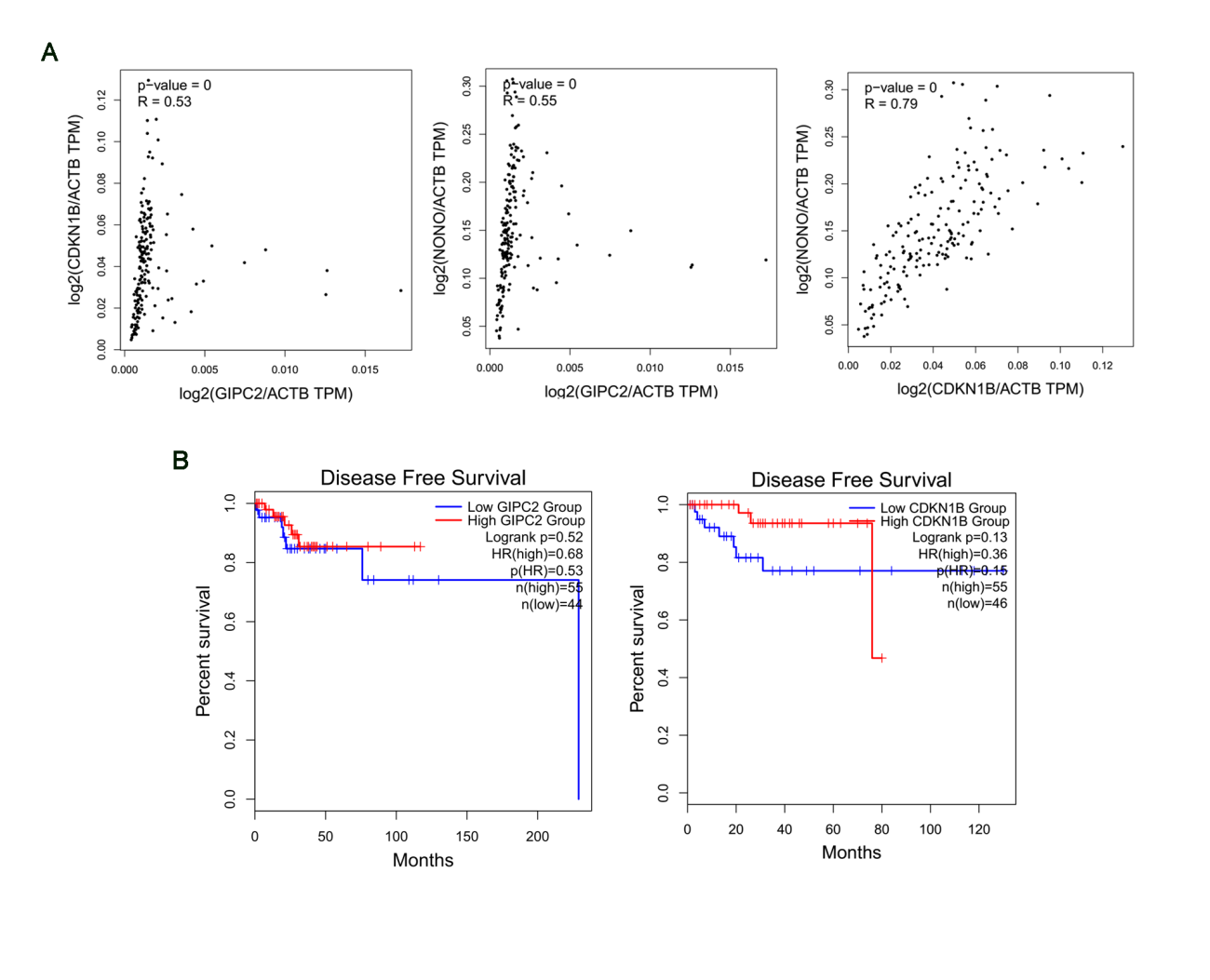
**

**Supplementary Fig.4 Clinicopathological relevance of *GIPC2* and *CDKN1B* (p27) in PPGL.**

**A** The correlations in mRNA expression between *GIPC2* and *CDKN1B* (*p27*), *GIPC2* and *NONO*, *and CDKN1B* and *NONO*. The relative level of *GIPC2*, *NONO* and *CDKN1B* was plotted against each other. B: Kaplan–Meier survival analysis for TCGA-PCPG dataset for the relationship between disease-free survival time and the *GIPC2* or *CDKN1B* signature.


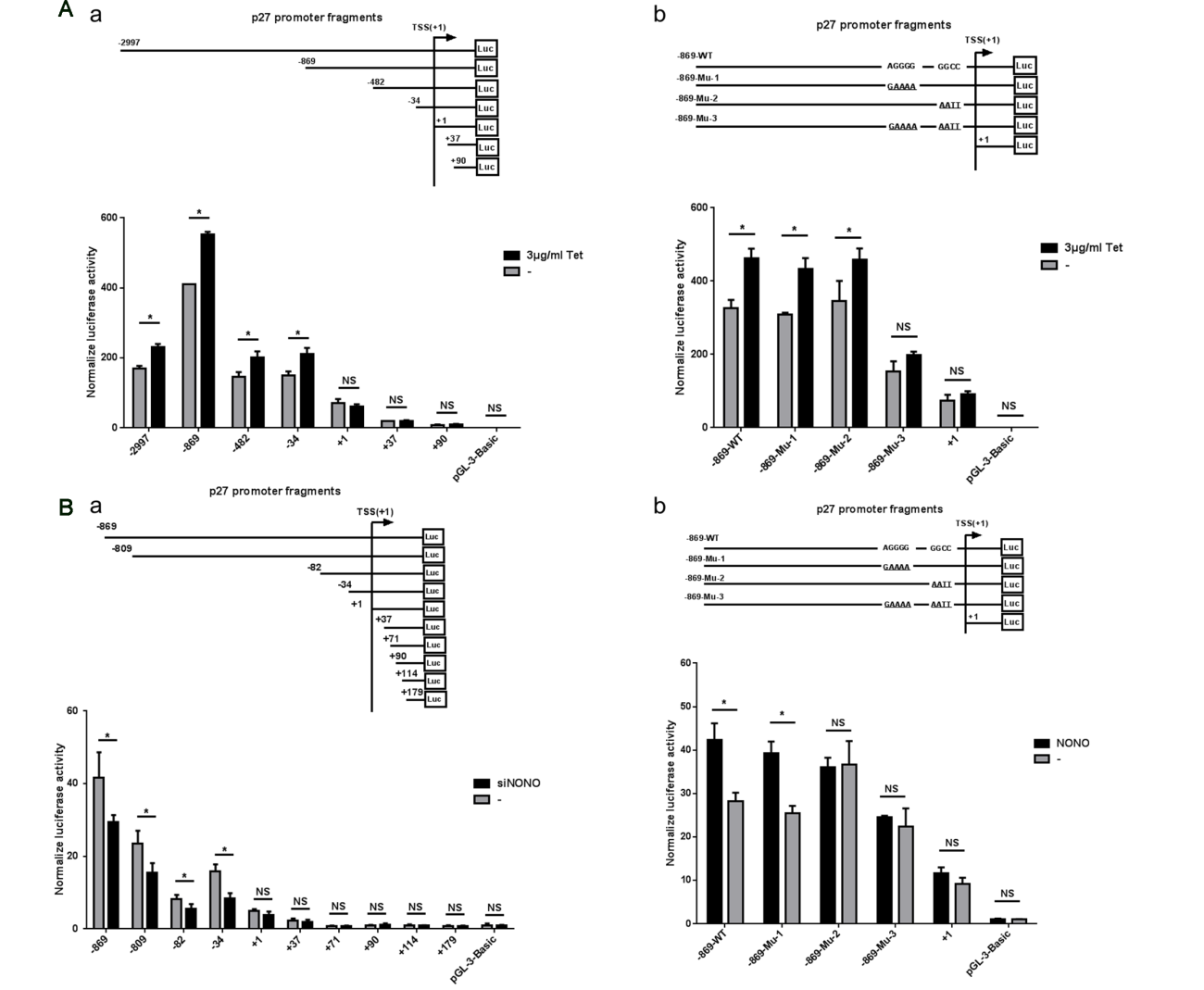


**Supplementary Fig.5 Identification of the p27promoter binding sites of GIPC2 and NONO, related to Fig 4.**

**A** hPheo1 cells with Tet-on lentiviruses systems carrying GIPC2 were transfected with the constructs of the truncated fragments of *p27* promoter luciferase plasmids (**a)** or mutants plasmids (**b)** after 6h treated with tetracycline (3μg/mL) to induce GIPC2 overexpression. 48h later, luciferase activity was measured. **B** hPheo1 cells were transfected with si-NONO or control, together with the constructs of the truncated fragments of *p27* promoter luciferase plasmids (**a)** or mutant plasmids (**b)** 48h later, luciferase activity was measured. Relative luciferase activity was calculated as firefly luciferase activity divided by renilla luciferase activity and shown relative to the control (transfected with pGL-3-Basic vector).


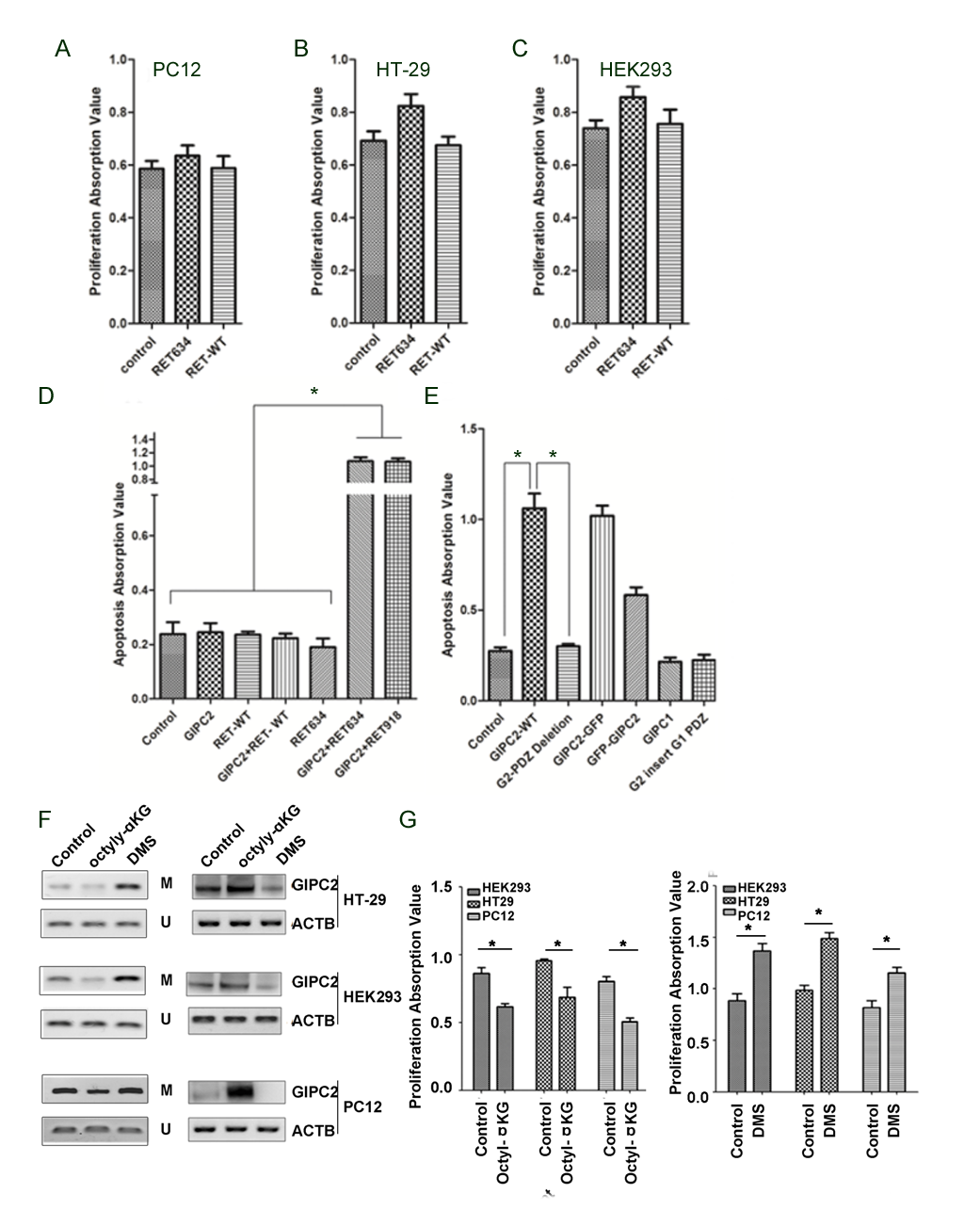


**Supplementary Fig.6 *RET* mutations lead to cell proliferation in the presence of 10** μ**M dexamethasone (Dex) but induced apoptosis when co-expressed with *GIPC2* in the absence of Dex.**

The proliferation of PC12(**A**), HT-29(**B**), and HEK293(**C**) when transfected with RET634 or RET-WT in the presence of 10 μM Dex. **D** The apoptosis assay of PC12 cells transfected with indicated constructs in the absence of Dex treatment. **E** PC 12 cells were first transfected with RET 634 mutant. After 24 hours, different GIPC2 variants or GIPC1 were transfected into these cells, and 48h later, apoptosis was assayed using Cell Death Detection ELISA Kit.


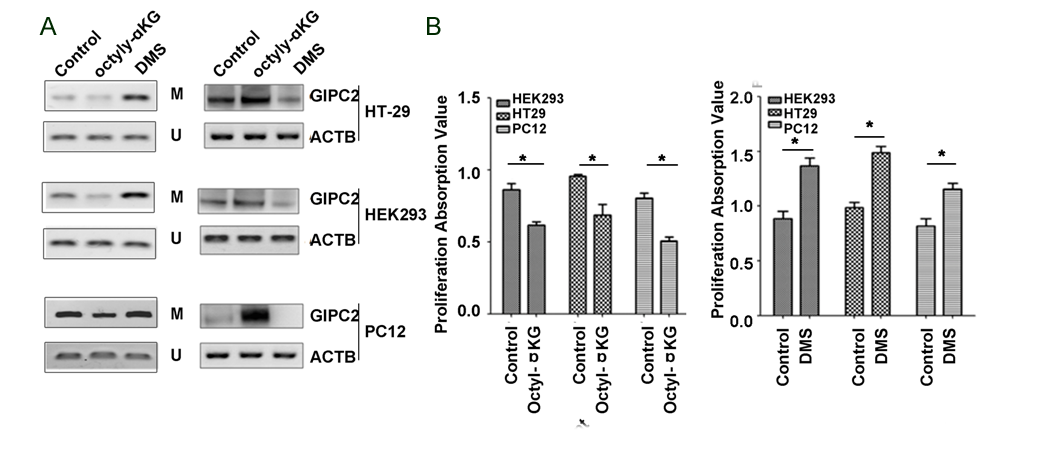


**Supplementary Fig.7 SDH inactivation promoted *GIPC2* promoter methylation and decreased its expression in HT-29，HKE293 and PC12*.***

**A** Methylation-specific PCR assay was carried out to detect the methylation of GIPC2 promoter CpG island on DNA isolated fromHT-29, HEK293 and PC12 cells treated with αKG or DMS. Corresponding GIPC2 protein levels by western blot were shown on the right. **B** The proliferation of HT-29, HEK293 and PC12 cells treated with αKG or DMS for 48 h.
