# Supplementary material for: GIPC2 is an endocrine-specific tumor suppressor gene for both sporadic and hereditary tumors of RET- and SDHB-, but not VHL-associated clusters of pheochromocytoma/paraganglioma
Source: Cell Death Dis. 2021 May 4;12(5):444. doi: 10.1038/s41419-021-03731-7 (PMC8096975; doi:10.1038/s41419-021-03731-7)
Supplement: Supplementary file 2 — Supplementary Tables [file 41419_2021_3731_MOESM2_ESM.doc]

**Supplementary Table 1. 25** gene list screened by microarray and bioinformatics

| **Gene Symbol** | **Chromosomal Location** | **Fold Change** | **Deletion sample number of**  **22 cases** | **Deletion percentage**  **of 78 cases*** |
| --- | --- | --- | --- | --- |
| HSD3B2 | chr1p13.1 | -178.373 | 14 | 73.1% |
| PTGFRN | chr1p13.1 | -4.795 | 14 | 74.4% |
| ADORA3 | chr1p13.2 | -9.027 | 14 | 76.9% |
| LOC729970 | chr1p21.3 | -4.676 | 14 | 98.7% |
| TMEM56 | chr1p21.3 | -3.747 | 14 | 98.7% |
| DDAH1 | chr1p22 | -2.264 | 15 | 97.4% |
| MCOLN2 | chr1p22 | -2.857 | 15 | 97.4% |
| MCOLN3 | chr1p22.3 | -22.533 | 15 | 96.2% |
| WDR63 | chr1p22.3 | -8.137 | 15 | 96.2% |
| DBT | chr1p31 | -2.475 | 14 | 78.7% |
| DIRAS3 | chr1p31 | -10.731 | 14 | 89.7% |
| CTH | chr1p31.1 | -6.808 | 14 | 88.5% |
| GIPC2 | chr1p31.1 | -9.035 | 15 | 93.6% |
| HHLA3 | chr1p31.1 | -2.768 | 14 | 88.5% |
| SAMD13 | chr1p31.1 | -3.009 | 15 | 97.4% |
| CACHD1 | chr1p31.3 | -4.690 | 14 | 78.2% |
| TGFBR3 | chr1p33-p32 | -4.9 | 14 | 97.4% |
| AGTR1 | chr3q24 | -6.763 | 11 | 73.1% |
| CPB1 | chr3q24 | -40.923 | 11 | 73.1% |
| VEPH1 | chr3q24-q25 | -16.989 | 11 | 74.4% |
| AADAC | chr3q25.1 | -37.889 | 11 | 53.8% |
| PLD1 | chr3q26 | -4.194 | 12 | 60.3% |
| IGF2BP2 | chr3q27.2 | -3.571 | 11 | 76.9% |
| LEPREL1 | chr3q28 | -4.953 | 11 | 78.2% |
| CLDN1 | chr3q28-q29 | -12.073 | 11 | 78.2% |

*Using CNV results from GSE38525 database of 87 Cluster 2A PPGL samples, as described in reference 18.

**Supplementary Table 2.** Oligonucleotides used in this study

| **Oligonucleotides** | | **Sequence** |
| --- | --- | --- |
| -869-WT | CGAGGGGAGGTGGCGGAACCGCGCCGGGGCCACC | |
| -869-Mu-1 | CG**GAAAA**AGGTGGCGGAACCGCGCCGGGGCCACC | |
| -869-Mu-2 | CGAGGGGAGGTGGCGGAACCGCGCCGG**AATT**ACC | |
| -869-Mu-3 | CG**GAAAA**AGGTGGCGGAACCGCGCCGG**AATT**ACC | |

The mutant sequences are bolded and underlined.

**Supplementary Table 3.** Primers used in this study

| **Primer** | **Sequence(5’-3’)** | **Purpose** |
| --- | --- | --- |
| p27 pro-2997-F^1^ | CTAGCTAGCAGGACTGAAACTGTGTGCTTGCG | -2997 |
| p27 pro-869-F | CTAGCTAGCCGGCTGGACTCAGGTAGAGGAAAC | -869 |
| p27 pro-482-F | CTAGCTAGCGCGAACCATTGCCCACTGC | -482 |
| p27 pro-82-F | CTAGCTAGCGACCAGCCAATCTCCCGG | -82 |
| p27 pro-34-F | CTAGCTAGCCGAGGGGAGGTGGCGGAAC | -34 |
| p27 pro+1-F | CTAGCTAGCTTAAGGCCGCGCTCGCCA | +1 |
| p27 pro+37-F | CTAGCTAGCCCGCCGCCGCAACCAATG | +37 |
| p27 pro+71-F | CTAGCTAGCTAAATAGACTCGCCGTGTCAATCAT | +71 |
| p27 pro+90-F | CTAGCTAGCAATCATTTTCTTCTTCGTCAGCCTC | +90 |
| p27 pro+114-F | CTAGCTAGCCCCTTCCACCGCCATATTG | +114 |
| p27 pro+179-F | CTAGCTAGCCCTCCCCTGTCCCCGCTTG | +179 |
| p27 pro+328-R^2^ | CCCAAGCTTGAGGGGACCAGGCAAGCGG | Luciferase reporters |
| p27 pro-869-Mu-1-F | GGAACGGAAAAAGGTGGCGGAACCGCGCCGGG | -869-Mu-1 |
| p27 pro-869-Mu-1-R | CCACCTTTTTCCGTTCCCGAGCGCGCCGCCTC | -869-Mu-1 |
| p27 pro-869-Mu-2-F | GCGCCGGAATTACCTTAAGGCCGCGCTCGCCAG | -869-Mu-2 |
| p27 pro-869-Mu-2-R | TTAAGGTAATTCCGGCGCGGTTCCGCCACCTCC | -869-Mu-2 |
| -869- -749-F | CGGCTGGACTCAGGTAGAGGAAA | ChIP -869- -749 |
| -869- -749-R | GGATCTTCCTTCCCAAGCACAGTTA | ChIP -869- -749 |
| -39- +99-F | CGGGAACGAGGGGAGGTG | ChIP -39- +99 |
| -39- +99-R | GAAAATGATTGACACGGCGAG | ChIP -39- +99 |
| GAPDH-F | ACCCAGAAGACTGTGGATGG | ChIP GAPDH |
| GAPDH-R | TTCAGCTCAGGGATGACCTT | ChIP GAPDH |

^1^ F: Forward.

^2^ R: Reverse.
