# Supplementary material for: GIPC2 is an endocrine-specific tumor suppressor gene for both sporadic and hereditary tumors of RET- and SDHB-, but not VHL-associated clusters of pheochromocytoma/paraganglioma
Source: Cell Death Dis. 2021 May 4;12(5):444. doi: 10.1038/s41419-021-03731-7 (PMC8096975; doi:10.1038/s41419-021-03731-7)
Supplement: Supplementary file 3 — Supple. File 1. Clinical information of the PPGL samples [file 41419_2021_3731_MOESM3_ESM.doc]

1. **Basic clinical information of the PPGL samples**

**Table 1. Clinical Characteristics of samples from 55 PPGL** Patients

| **Variable** | **Number or Mean±SD** |
| --- | --- |
| Age at diagnosis (yr) | 45 ±14 |
| Sex (M/F) | 30/25 |
| Benign/Malignant | 54/1 |
| Sporadic/Hereditary | 49/6 |
| Adrenal/Extraadrenal | 49/6 |
| Tumor diameter (cm) | 4.2±1.6 |

1. **Screening of known susceptibility gene mutations in samples**

All the coding regions of VHL, SDHB, SDHC, SDHD and RET exons 10, 11, 13, 14, 15 and 16 were sequenced with Sanger sequencing. Other genes were not screened due to their expected low frequency. Familial NF1 pheochromocytoma was excluded based on clinical criteria and family history. No mutations of VHL, SDHx were found in tumor samples, while germline mutation of RET was found in 6 samples and somatic mutation of RET was found in 1 sample (Table. 2, Fig. 1). In addition, we detected four SNPs of RET (rs1799939, rs11238441, rs2435353, rs1800863).

**Table 2. RET mutations in the tumors of patients**

| **ID** | **Family history** | **Age at Diagnosis** | **Tumor Type** | **Tumor Location** | **Mutations (nucleotide, protein)** | **Mutation type** |
| --- | --- | --- | --- | --- | --- | --- |
| 1652767 | N | 73 | MEN2/PCC | A | c.1902C>G  p.C634W | Germline Missense# |
| 1720848 | N | 13 | MEN2/PCC | A | c.1901G>A  p.C634Y | Germline Missense# |
| 1653937 | N | 49 | MEN2/PCC | A | c.1901G>A  p.C634Y | Germline Missense# |
| 1605301 | N | 44 | MEN2/PCC | A | c.1900T>C  p.C634R | Germline Missense# |
| 1508247 | N | 45 | MEN2/PCC | A | c.1894G>A  p.G632K | Germline Missense* |
| 1649892 | N | 40 | MEN2/PCC | A | c.1901G>A  p.C634Y | Germline Missense# |
| 1611591 | N | 55 | PCC | A | c.1902C>G  p.C634W | Somatic Missense# |

N = no; A = adrenal; PCC=pheochromocytoma; MEN2=Multiple Endocrine Neoplasia type 2; # known mutation; *novel mutation


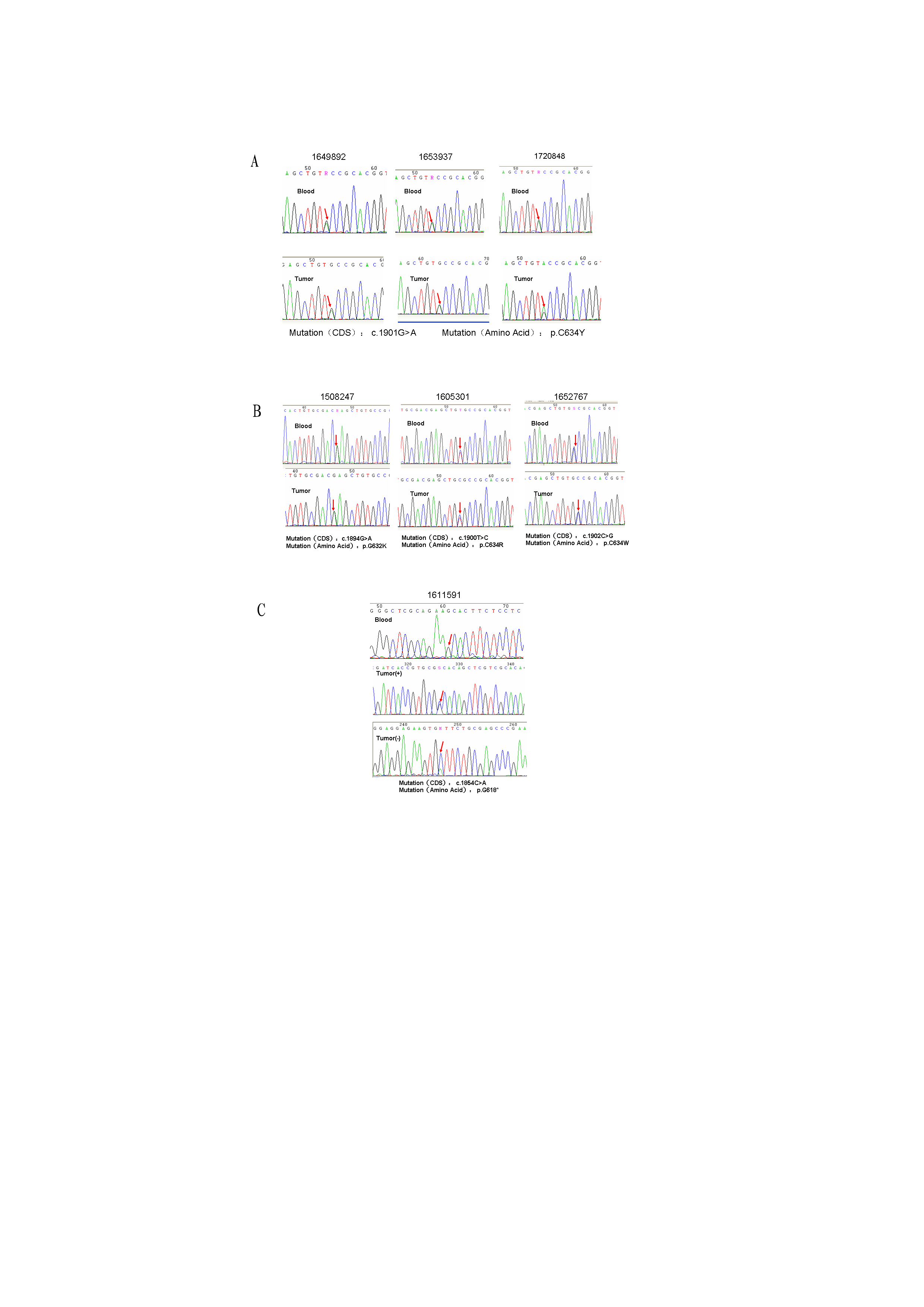


**Figure. 1 RET mutation by Sanger sequencing**
